# *Mycobacterium tuberculosis* Drug Resistance in Ethiopia: An Updated Systematic Review and Meta-Analysis

**DOI:** 10.3390/tropicalmed7100300

**Published:** 2022-10-14

**Authors:** Melese Abate Reta, Birhan Alemnew Tamene, Biruk Beletew Abate, Eric Mensah, Nontuthuko Excellent Maningi, P. Bernard Fourie

**Affiliations:** 1 Department of Medical Microbiology, Faculty of Health Sciences, University of Pretoria, Prinshof 0084, South Africa; 2 Department of Medical Laboratory Science, College of Health Sciences, Woldia University, Woldia P.O. Box 400, Ethiopia; 3 Department of Nursing, College of Health Sciences, Woldia University, Woldia P.O. Box 400, Ethiopia or; 4 Department of Microbiology, School of Life Sciences, College of Agriculture, Engineering and Science, University of Kwazulu Natal, Durban 4041, South Africa

**Keywords:** *Mycobacterium tuberculosis*, drug-resistance, MDR-TB, meta-analysis, Ethiopia

## Abstract

**Background:** Tuberculosis (TB) remains a significant global public health issue, despite advances in diagnostic technologies, substantial global efforts, and the availability of effective chemotherapies. *Mycobacterium tuberculosis*, a species of pathogenic bacteria resistant to currently available anti-TB drugs, is on the rise, threatening national and international TB-control efforts. This systematic review and meta-analysis aims to estimate the pooled prevalence of drug-resistant TB (DR-TB) in Ethiopia. **Materials** **and Methods:** A systematic literature search was undertaken using PubMed/MEDLINE, HINARI, the Web of Science, ScienceDirect electronic databases, and Google Scholar (1 January 2011 to 30 November 2020). After cleaning and sorting the records, the data were analyzed using STATA 11. The study outcomes revealed the weighted pooled prevalence of any anti-tuberculosis drug resistance, any isoniazid (INH) and rifampicin (RIF) resistance, monoresistance to INH and RIF, and multidrug-resistant TB (MDR-TB) in newly diagnosed and previously treated patients with TB. **Results:** A total of 24 studies with 18,908 patients with TB were included in the final analysis. The weighted pooled prevalence of any anti-TB drug resistance was 14.25% (95% confidence interval (CI): 7.05–21.44%)), whereas the pooled prevalence of any INH and RIF resistance was found in 15.62% (95%CI: 6.77–24.47%) and 9.75% (95%CI: 4.69–14.82%) of patients with TB, respectively. The pooled prevalence for INH and RIF-monoresistance was 6.23% (95%CI: 4.44–8.02%) and 2.33% (95%CI: 1.00–3.66%), respectively. MDR-TB was detected in 2.64% (95%CI: 1.46–3.82%) of newly diagnosed cases and 11.54% (95%CI: 2.12–20.96%) of retreated patients with TB, while the overall pooled prevalence of MDR-TB was 10.78% (95%CI: 4.74–16.83%). **Conclusions:** In Ethiopia, anti-tuberculosis drug resistance is widespread. The estimated pooled prevalence of INH and RIF-monoresistance rates were significantly higher in this review than in previous reports. Moreover, MDR-TB in newly diagnosed cases remained strong. Thus, early detection of TB cases, drug-resistance testing, proper and timely treatment, and diligent follow-up of TB patients all contribute to the improvement of DR-TB management and prevention. Besides this, we urge that a robust, routine laboratory-based drug-resistance surveillance system be implemented in the country.

## 1. Introduction

Tuberculosis (TB) has been known as a human pathogen for decades and is still a significant global health problem [1,2,3]. Despite advances in diagnostic tools, the availability of effective anti-TB therapy, and substantial global efforts, about 10.0 million people contracted TB in 2019, resulting in over 1.4 million deaths [1]. The burden of national TB epidemics varies significantly between countries, with Asia and Africa being the most affected continents [1]. Due to a multitude of causes, TB has the highest mortality rate of all infectious diseases in “low- and middle-income countries” (LMICs). Poor access to healthcare facilities, weak TB preventive and control systems, overcrowded living conditions, occupational hazards, individuals’ poor nutritional status, a high prevalence of human immunodeficiency virus (HIV), and other comorbidities and drug addiction all lead to the high incidence of TB in LMICs [4,5]. Ethiopia, like other LMICs, is experiencing an increase in TB incidence since 2001, posing major challenges for the public healthcare system and national TB-control efforts [3].

Drug-resistant tuberculosis (DR-TB) strains, particularly multidrug-resistant TB (MDR-TB), continue to pose a serious threat to public healthcare systems, mainly in resource-constrained nations such as Ethiopia, where innovative molecular diagnostic technologies and well-equipped laboratory settings are lacking [3,6,7]. DR-TB usually occurs due to the patient’s delay in early diagnosis and treatment, previous anti-TB drug exposure [8], inappropriate drug regimens [9], the patient’s poor adherence to anti-tuberculosis drug regimens [10], and primary infection with DR-TB strains [11,12,13]. Antibiotic resistance develops in *Mycobacterium tuberculosis* strains due to spontaneous gene alterations that decrease the susceptibility of the bacterium to the most widely used anti-tuberculosis drugs. These genes can encode drug targets or mechanisms of drug metabolism, affecting the efficacy of anti-TB therapy [1,14,15]. Failure to diagnose drug-resistance and subsequent improper therapy of TB-patient increase the risk of developing drug resistance and the direct transmission of DR-TB strains to other individuals [1,14]. Treatment of DR-TB, particularly MDR-TB, is time-consuming and costly, requiring the use of second-line anti-TB medicines that are more toxic and ineffective [1,3].

In resource-constrained settings such as Ethiopia, DR-TB, particularly MDR-TB detection rate, is under-reported due to a lack of reliable, quick, and affordable diagnostic testing. Nearly half a million people worldwide contracted RIF-resistant TB (RR-TB) in 2019, with 78.0% of those suffering from MDR-TB [1]. According to the recent World Health Organization (WHO) 2019 report, MDR/RR-TB was found in 3.4% of newly tested and 18.0% of patients previously treated with TB worldwide [3]. Ethiopia is among the top 30 countries with the highest rates of TB, DR-TB, and TB/HIV co-infection [3]. According to a recent national report, Ethiopia’s TB incidence was reported to be 157 per 100,000 people, with 23,800 people dying from the disease [1]. In Ethiopia, MDR-TB was found in 0.71% of newly diagnosed cases and 12.0% of patients previously treated with TB in 2019 [1].

To effectively treat and prevent the spread of DR-TB strains, all patients with TB must undergo anti-TB drug susceptibility testing (DST), either by standard phenotypic or by using effective and rapid molecular diagnostic methods [3,16]. However, mycobacterial culture on a liquid or solid medium and conventional DST is time-consuming and requires well-equipped laboratory settings and extensive biosafety resources to obtain results that inform the initiation of proper anti-TB drug treatment. This is impractical in several resource-constrained countries, including Ethiopia [1,15,17]. Moreover, phenotypic DST methods usually lack accuracy and reproducibility [17]. For these reasons, the use of reliable and rapid molecular diagnostic methods is recommended and widely practiced globally. Molecular testing assays, such as the “GeneXpert^®^MTB/RIF and Ultra assays” (“Cepheid, Sunnyvale, CA, USA”) and the line probe assays “GenoType^®^MTBDR*plus*” and “GenoType^®^MTBDR*sl*” (“Hain Life-science GmbH, Nehren, Germany”), have been shown to significantly reduce the time required to initiate therapy in patients with TB and DR-TB and for the establishment of the appropriate treatment regimen [1]. GeneXpert is the best available quick diagnostic test since it simultaneously detects *M. tuberculosis* and the RIF resistance gene. The negative predictive value and accuracy of GeneXpert were found to be superior to “acid-fast bacilli” smear microscopy [18]. The “Xpert MTB/XDR”, the other recently developed molecular diagnostic assay, provides high accuracy for the diagnosis of INH and FLQ resistance and can support the choice of the appropriate treatment regimen for TB patients [19]. Similarly, “ a broad-range PCR coupled with electrospray ionization mass spectrometry” offers an alternative to existing diagnostic techniques for the rapid detection of genetic markers in INH and RIF resistance in *M. tuberculosis* strains [20].

Following the recent WHO recommendation to use GeneXpert and line probe assays, which are effective and rapid molecular testing technologies, as part of an initial TB diagnostic test [3], there has been a steady increase in the reporting of high numbers of detected TB and DR-TB cases in developing countries, including Ethiopia [3,6,13,21]. Ethiopia’s Federal Ministry of Health has rolled out the GeneXpert^®^MTB/RIF (“Cepheid, Sunnyvale, CA, USA”) molecular diagnostic assay into the country’s current national TB diagnostic system, with line probe assay performed in each regional referral laboratory center, resulting in increased TB case detection, early identification of DR-TB isolates, and the provision of effective and appropriate therapy of TB patients [3,22]. Thus, the rate of TB and DR-TB case detection at the national level is improving [1,23]. Several previous systematic reviews and meta-analysis studies undertaken in Ethiopia have estimated the pooled prevalence of DR-TB using phenotypic DST data or data derived from both phenotypic and molecular DST findings [24,25,26,27]. However, both phenotypic and molecular DST methods have different degrees of sensitivity and specificity, and many factors can affect the accuracy of DST performance. Given the paucity of data on the weighted pooled prevalence of anti-tuberculosis drug resistance using molecular diagnostic laboratory results, the main aim of this meta-analysis is to provide an up-to-date data analysis on the weighted pooled prevalence of any anti-TB drug-resistance, any isoniazid (INH) and rifampicin (RIF) resistance, monoresistance to INH and RIF, and MDR-TB, in newly diagnosed TB cases and retreated patients with TB in Ethiopia. This systematic review and meta-analysis provides relevant data to better understand the magnitude of DR-TB and helps the country to pursue evidence-based measures for DR-TB control and to establish robust and routine laboratory-based DR-TB surveillance using affordable and rapid molecular diagnostic methods.

## 2. Materials and Methods

### 2.1. Study Protocol

The search strategy for potential articles, screening them by title and abstract, and evaluating their eligibility to be included in the final analysis, was conducted using the “Preferred Reporting Items for Systematic Reviews and Meta-Analysis” Protocol [28], (Supplementary Table S1). This review protocol has been submitted to and registered in the “International Prospective Register of Systematic Reviews” (ID#: CRD42020176713) (https://www.crd.york.ac.uk/PROSPERO/display_record.php?RecordID=176713) (accessed on 12 March 2022).

### 2.2. Databases and Search Strategy

PubMed/MEDLINE, HINARI, Web of Science, Science Direct electronics databases, and Google Scholar were searched for relevant articles published in English (1 January 2011 to 30 November 2020). We excluded non-English language papers due to a lack of language resources (e.g., professional translators). The following search terms were used: “*Mycobacterium tuberculosis*”, “tuberculosis”, “drug-resistance”, “drug susceptibility testing”, “mono-resistance”, “anti-TB drug-resistance”, “DR-TB”, “MDR/RR-TB”, “isoniazid-resistant tuberculosis”, “rifampicin-resistant tuberculosis”, “ethambutol-resistant tuberculosis”, “pyrazinamide-resistant tuberculosis”, “molecular diagnostics”, “molecular detection”, “molecular characterization”, “Line Probe Assay”, “GenoType^®^MTBDR*plus* assay”, “GenoType^®^MTBDR*sl* assay”, “XpertMTB/RIF”, “GeneXpert^®^MTB/RIF assay”, and “Ethiopia”. The search strings were applied using “AND” and “OR” Boolean operators. The complete search strategy for the PubMed/MEDLINE database is provided as a supplementary file (Supplementary Table S2). Besides this, we reviewed the reference lists of primary studies and review articles to gain access to grey literature.

### 2.3. Selection Criteria and Data Extraction

The records found through database searching were merged and the duplicates were removed using EndNote X7 (“Thomson Reuters, New York, NY, USA”). Three reviewers (screeners 1, 2, and 3) screened all titles and abstracts independently and excluded irrelevant data, then independently assessed the remaining articles for inclusion. The information extracted from each study included (a) author’s name, (b) publication year, (c) study period, (d) study area, (e) types of patients with TB (pulmonary TB and extra-pulmonary TB), (f) study design, (g) molecular diagnostic methods (LPA, sequencing, and other PCR-based), (h) type of TB cases (newly diagnosed and retreated), (i) the number of patients, (j) total TB positive cases, (k) total patients/isolates with available DST results, (l) any drug-resistance, (m) monoresistance, and (*n*) MDR-TB cases in newly diagnosed TB cases and retreated patients with TB (Table 1).

Studies that addressed any of the following criteria were included: (a) studies that used WHO-approved molecular DST methods; (b) studies that reported data on the prevalence of drug-resistance in retreated patients with TB or newly diagnosed TB cases [among both pulmonary TB (PTB) and extra-pulmonary TB (EPTB) patients]; (c) studies reporting the prevalence of any anti-tuberculosis drug-resistance, monoresistance, or MDR/or extensively drug-resistant-TB (XDR-TB); (d) studies conducted in Ethiopia and published in the English language. Whereas studies were excluded if they met the following conditions: (a) studies with non-tuberculous mycobacteria data; (b) studies that did not perform DST of first- and second-line anti-TB drugs; (c) studies that only used phenotypic DST methods to detect DR-TB. This study also excluded the following: editorial papers, narrative reviews, meta-analysis and/or systematic reviews, conference abstracts, and case reports. Qualitative studies and citations without full text were also excluded.

### 2.4. Quality Assessment

Two writers (MAR and BAT) independently evaluated the quality of the included studies using an updated version of the tool proposed by the “Joanna Briggs Institute (JBI)” [29]. A third author (BBA) acted as an arbiter and adjudicated in any cases where there was disagreement. Studies (case-control, cross-sectional, and cohort) with an average score of four or higher were considered high quality and included, whereas studies with an average score of three or lower were considered low quality and excluded (Supplementary Table S3).

### 2.5. Definitions

The following standard definitions of anti-TB drug resistance were used [2,30]. (a) “Any drug-resistance”: *M. tuberculosis* strains resistant to one or more first-line anti-TB drugs, regardless of monoresistance or MDR-TB; (b) “Any INH-resistance”: INH-monoresistance, INH hetero-resistance, and/or MDR-TB patients; (c) “Any RIF-resistance”: referred to as RIF-monoresistance, RIF hetero-resistance, and/or MDR-TB patients; (d) “INH monoresistance”: TB infection caused by *M. tuberculosis* strains resistant to INH only; (e) “RIF monoresistance”: TB infection caused by *M. tuberculosis* strains resistant to RIF only; (f) “MDR-TB”: *M. tuberculosis* strains resistant to at least INH and RIF; (g) “MDR-TB among new TB cases”: *M. tuberculosis* isolates resistant to both INH and RIF in patients with TB who have never received treatment for TB; (h) “MDR-TB among retreated patients with TB”: *M. tuberculosis* isolates resistant to INH and RIF in patients who have been received treatment for TB; (i) “Molecular DST techniques”: DST methods that use WHO-certified nucleic acid amplification technologies (NAATs) to diagnose DR-TB, such as LPA, sequencing, and other PCR-based methods.

**Table 1:** Characteristics of included studies.

Author/s	Year of Publication	Study Period	Study Region	Type of Patients	Study Design	Molecular Diagnostic Methods	Type of TB Cases		Total Number of Patients (*n*)	Total Positive Cases (*n*)	Total Isolates with Available DST Results (*n*)	Any Drug Resistance (*n*)	Any Anti-TB Drug Resistance, (*n*)				Mono-Resistance, (*n*)			MDR, (*n*)		
																				Retreated Cases	New Cases	Overall
							New (*n*)	Retreated (*n*)					INH, (*n*)	RIF, (*n*)	EMB, (*n*)	FLQ, (n)	INH, (*n*)	RIF, (*n*)	EMB, (*n*)			
Zewdie et al. [31]	2018	2014	AA	EPTB	Cross-sectional	GenoType^®^MTBDR*plus*	52	8	65	60	60	6	6	5	NS	NS	1	0	NS	3	2	5
Workalemahu et al. [32]	2013	2011	OR	PTB	NR	GenoType^®^MTBR*plus*	NR	NR	121	15	15	1	1	NR	NS	NS	1	0	NS	0	0	0
Wondale et al. [33]	2018	2014–2016	SNNP	PTB and EPTB	NR	GenoType^®^MTBDR*plus* V.2.	153	8	161	126	126	4	1	3	NS	NS	NR	2	NS	1	0	1
Tessema et al. [34]	2012	2009	AM	PTB	Cross-sectional	GenoType^®^MTBDR*plus* and GenoType^®^MTBDR*sl*	214	46	260	260	260	45	35	15	8	NR	22	2	NR	5	8	13
Tadesse et al. [35]	2016	2013–2014	OR	PTB	Cross-sectional	GenoType^®^MTBDR*plus* V.2	41	71	122	118	112	44	41	34	NS	NS	10	3	NS	26	5	31
Tadesse et al. [36]	2017	2013–2015	OR	EPTB	NR	GeneXpertMTB/RIF	NR	NR	436	310	279	10	NS	10	NS	NS	NS	NR	NS	NR	NR	NR
Sinshaw et al. [37]	2019	2017–2018	AA	PTB	Cross-sectional	GenoType^®^MTBDR*plus* V.2 and Genotype^®^MTBDR*sl* V.2	345	73	418	26	26	10	10	3	NR	0	7	0	NR	1	2	3
Mulu et al. [38]	2017	2014–2015	AM	PTB and EPTB	Cross-sectional	GeneXpertMTB/RIF	373	132	505	117	117	12	NS	12	NS	NS	NS	NS	NS	NR	NR	NR
Jaleta et al. [39]	2017	2013–2015	AM	PTB	Cross-sectional (Retro)	GeneXpert MTB/RIF	305	1515	1820	448	448	71	NS	71	NS	NS	NS	NS	NS	NR	NR	NR
Haile et al. [40]	2020	2015–2016	OR	PTB	NR	GenoType^®^MTBDR*plus*	105	6	111	92	92	6	5	1	NS	NS	5	1	NS	0	0	0
Habte et al. [41]	2016	2013–2015	AM	PTB	Cross-sectional	GeneXpert MTB/RIF	119	0	119	111	111	5	NS	5	NS	NS	NS	NR	NS	NR	NR	NR
Gizachew Beza et al. [42]	2017	2016	AM	PTB	Cross-sectional	GeneXpert MTB/RIF	243	22	265	9	9	1	NS	1	NS	NS	NS	NR	NS	NR	NR	NR
Gebrehiwet et al. [43]	2019	2016–2017	AF	PTB	Cross-sectional	GeneXpertMTB/RIF	321	63	384	94	94	4	NS	4	NS	NS	NS	NR	NS	NR	NR	NR
Fanosie et al. [44]	2016	2015	AM	PTB	Cross-sectional	GenoType^®^MTBDR*plus* and GeneXpertMTB/RIF	141	NR	141	37	37	1	1	1	NS	NS	NR	NR	NS	NR	NR	1
Ejeta et al. [45]	2018	2017	GA	PTB	Cross-sectional	GeneXpertMTB/RIF	465	530	995	193	193	9	NS	9	NS	NS	NS	NR	NS	NR	NR	NR
Diriba et al. [46]	2019	2017–2018	Tig, HR, SNNP, AA, AM, OR	PTB	Cross-sectional	GenoType^®^MTBDR*plus*	NR	NR	10,134	1183	329	40	19	21	NS	NS	19	22	NS	22	16	38
Damena et al. [47]	2019	2015–2016	AA	PTB	Cross-sectional	GenoType^®^MTBDR*plus* V.2 and<break/>GenoType^®^MTBDR*sl*	98	115	213	150	150	20	20	16	12	1	4	0	0	11	5	16
Brhane et al. [48]	2017	NR	SO	PTB	Cross-sectional	GenoType^®^MTBDR*plus* V.2	67	31	105	98	98	18	18	10	NS	NS	8	0	NS	7	3	10
Biadglegne et al. [49]	2013	2012	AM	EPTB	Cross-sectional	GenoType^®^MTBDR*plus* and GenoType^®^ MTBDR*sl*	213	13	226	226	226	13	8	3	2	NS	6	1	NR	0	2	2
Biadglegne et al. [50]	2014	NR	AM	EPTB	Cross-sectional	GeneXpertMTB/RIF & GenoType^®^MTBDR*plus*	231	0	231	32	32	3	NS	3	NS	NS	NS	NR	NS	NR	NR	NR
Bekele et al. [51]	2018	2006–2010	AA, AM,<break/>OR, SNNPR	PTB & TBLN	Cross-sectional	GenoType^®^MTBDR*plus*	NR	NR	950	161	161	14	12	7	NS	NS	7	2	NS	NR	NR	5
Bedewi Omer et al. [52]	2016	2012–2013	OR	PTB	Cross-sectional	GenoType^®^MTBDR*plus*	268	11	279	279	279	31	25	9	NS	Ns	22	6	NS	0	3	3
Amir Alelign et al. [53]	2019	2015–2017	AM	PTB & EPTB	Cross-sectional	GenoType^®^MTBDR*plus*	90	21	111	111	111	20	20	2	NS	NS	18	0	NS	2	0	2
Abate et al. [54]	2014	2012–2013	AA	PTB	Cross-sectional (Retro)	GenoType^®^MTBDR*plus*	0	736	736	736	736	523	481	470	NS	NS	54	42	NS	427	0	427
Total							3844	3401	18,908	4992	4101	911	703	715	22	1	184	81	0	505	46	557

**Abbreviations:** TB, tuberculosis; DST, drug susceptibility testing; MDR, Multidrug resistance; INH, isoniazid; RIF, rifampicin; EMB, ethambutol; FLQ, fluoroquinolones; AA, Addis Ababa; PTB, pulmonary tuberculosis; Tig: Tigray; NS, not studied; AM, Amhara; EPTB, extra-pulmonary tuberculosis; OR, Oromia; SNNP, Southern Nation, Nationality, and Peoples; TBLN, Tuberculous lymphadenitis; SO, Somalia; HR, Harari; GA, Gambella; AF, Afar; NR: Note reported. MTBDR*plus* or MTBDR*sl* indicate the earlier versions of first and second-line Line Probe Assays, repectively.

### 2.6. Statistical Analysis

Important data from included studies were recorded/extracted using a standard data extraction format prepared in a Microsoft Excel spreadsheet and then exported to Stata/SE software (version 11, “StataCorp, College Station, TX, USA”) for final analysis. A random-effects model was used in the meta-analysis due to the heterogeneity of the studies. The heterogeneity of included studies was evaluated using Cochran’s Q test and the I^2^ index. The presence of studies’ publication bias was assessed utilizing Begg’s and Egger’s tests, and funnel plots of the standard error of the Logit event rate were provided to show the presence of publication bias. All statistical results and interpretations were reported with a 95% confidence interval (CI) basis. A statistical test with a *p* < 0.05 level of significance was considered statistically significant.

### 2.7. Study Outcomes

The main outcomes of the review were the weighted pooled prevalence of any anti-tuberculosis drug resistance, any resistance to INH and RIF, monoresistance to INH and RIF, and MDR-TB among newly diagnosed cases, retreated patients with TB, and overall TB cases.

## 3. Results

### 3.1. Search Results

**Figure 1:**
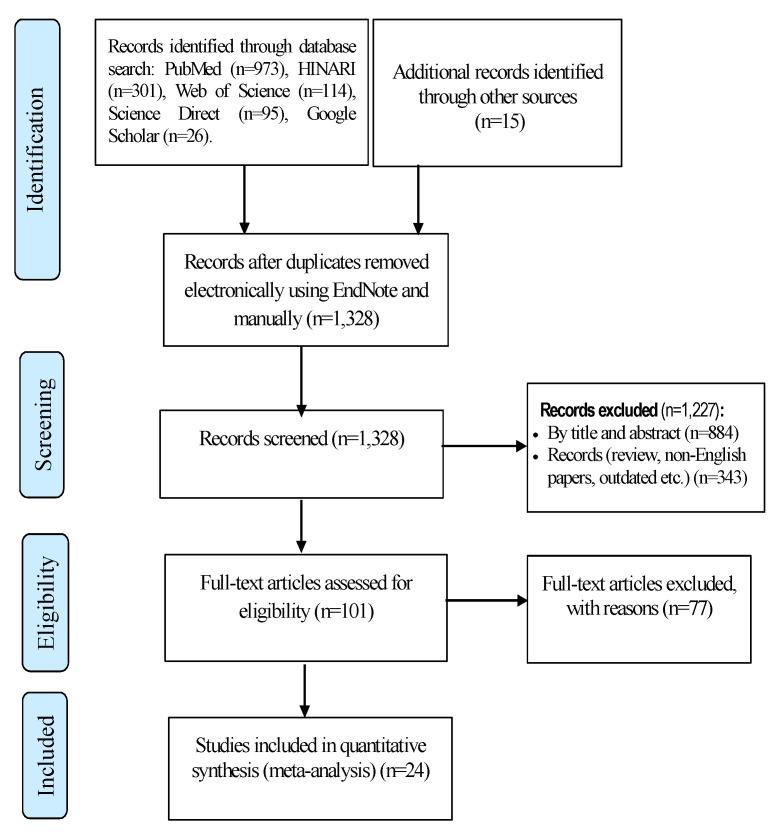
Flow chart of the study identification via databases, results of the search, and reasons for exclusion of articles.

Figure 1 shows a total of 1524 relevant studies recorded from searched electronic databases. Of the total, 1328 studies were non-duplicated and subjected to further evaluation; 1227 were assessed and excluded based on their title, abstract, and for other reasons (review, non-English papers, outdated, etc.), while 101 papers were retained for full-text review. After full-text evaluation, the final analysis (meta-analysis) included 24 articles that reported on drug-resistant *M. tuberculosis* isolates.

### 3.2. Characteristics of Included Studies

Twenty-four potential studies with a total of 18,908 patients with TB (PTB, *n* = 16,223; EPTB, *n* = 958; and PTB + EPTB, *n* = 1727) were included in the final analysis [31,32,33,34,35,36,37,38,39,40,41,42,43,44,45,46,47,48,49,50,51,52,53,54]. The types of patients with TB (new and retreated cases) were 3844 and 3401, respectively, while few studies did not mention the types of patients with TB. However, the rate of drug resistance was evaluated in 4101 *M. tuberculosis* isolates with available DST results in the included studies. In total, 911 *M. tuberculosis* isolates resistant to any anti-TB drug were identified in those included studies. The rate of MDR-TB was estimated using data from 16 studies comprising a total of 2818 *M. tuberculosis* isolates with complete DST profiles [31,32,33,34,35,37,40,44,46,47,48,49,51,52,53,54]. On the other hand, a total of 2818 *M. tuberculosis* isolates with complete DST results reported in 16 studies were examined for the occurrence of any INH resistance [31,32,33,34,35,37,40,44,46,47,48,49,51,52,53,54]. Most of the studies included in this review evaluated the occurrence of any RIF resistance, and we estimated the weighted pooled prevalence of any RIF resistance among a total of 4086 *M. tuberculosis* isolates [31,33,34,35,36,37,38,39,40,41,42,43,44,45,46,47,48,49,50,51,52,53,54]. INH-monoresistance [31,32,34,35,37,40,46,47,48,49,51,52,53,54], and RIF-monoresistance [31,32,33,34,35,37,40,46,47,48,49,51,52,53,54] were evaluated among 2655 and 2781 *M. tuberculosis* isolates with available DST results, respectively. Three studies [34,47,49] reported ethambutol-resistant TB (*n* = 22), whereas one study [47] identified fluoroquinolone-resistant TB (*n* = 1). The majority of studies (*n* = 9) were conducted in the Amhara region, followed by the Oromia Region (*n* = 5), and central Ethiopia, Addis Ababa (*n* = 4). Table 1 shows different versions of the assays, need to mention that these were also covered. It applies to GenoType assays (versions 1 and 2) and GeneXpert versions (MTB/RIF, Ultra).

### 3.3. Meta-Analysis Results

#### 3.3.1. Prevalence of Any Anti-TB Drug Resistance

**Figure 2:**
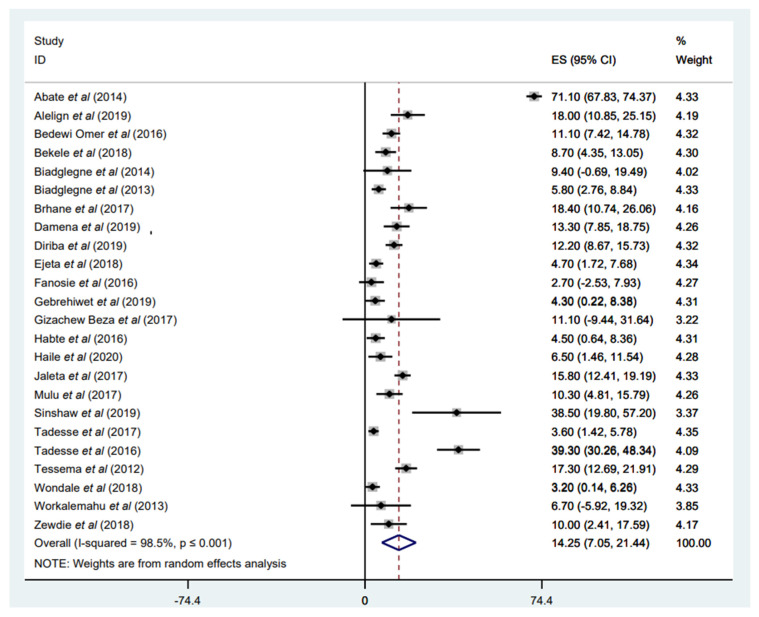
Forest plot showing the weighted pooled prevalence of any anti-TB drug resistance in patients with TB.

Any anti-tuberculosis drug resistance was reported in 24 studies, with a weighted pooled prevalence of 14.25% (95% CI: 7.05–21.44%) among 4101 *M. tuberculosis* isolates with available DST results; a substantial heterogeneity was (I^2^ = 98.5%) (Figure 2). The funnel plot, as well as Egger’s test result, which indicates a publication bias, and the sensitivity analysis of the included papers, are all presented in a supplementary file (Supplementary Figures S1A–E). Seven of the included studies reported the highest proportion of any anti-TB drug resistance, ranging from 15.80% to 71.10% [34,35,37,39,48,53,54], and three of them reported from the Amhara region [34,39,53]. The highest prevalence of any anti-TB drug resistance, with 71.10%, was reported by Abate et al. in central Ethiopia, Addis Ababa [54], while the lowest proportion, with 2.70%, was reported by Fanosie et al. in the Amhara region [44]. The high rate of any DR-TB observed by Abate et al. [54] could be due to the study’s inclusion of exclusively retreated patients with TB. Hence, the occurrence of DR-TB is anticipated to be prominent among retreated patients with TB (Figure 2).

#### 3.3.2. Prevalence of Any INH Resistance

**Figure 3:**
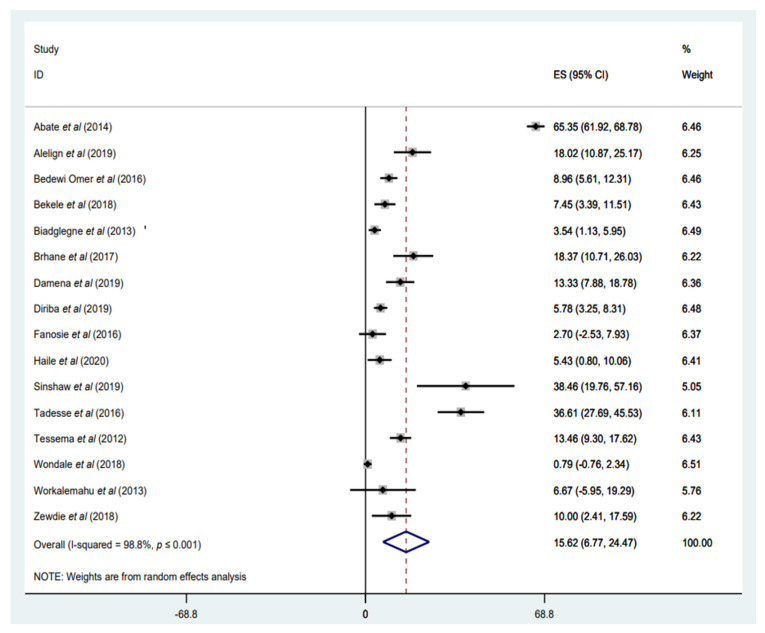
Forest plot showing the weighted pooled prevalence of any isoniazid (INH) resistance in tuberculosis (TB) patients.

Any INH resistance was reported in 16 studies, with a weighted pooled prevalence of 15.62% (95%CI: 6.77–24.47%; I^2^ = 98.8%) among a total of 2818 *M. tuberculosis* isolates (Figure 3). The funnel plot, as well as Egger’s test result, which indicates a publication bias, and the sensitivity analysis of the included papers, are all presented in the supplemental file (Supplementary Figures S2A–D). Geographically, the highest proportion of any INH resistance was recorded in seven studies, with rates ranging from 13.33% to 65.35% [34,35,37,47,48,53,54]; three of the studies were conducted in central Ethiopia, Addis Ababa [37,47,54], while two studies were conducted in the Amhara region [34,53]. The highest prevalence of any-INH resistance, with 65.35%, was reported in Addis Ababa [54]. This may be because all patients with TB in the study done by Abate et al. (2014) had been previously treated, which increases the likelihood of developing any INH resistance.

#### 3.3.3. Prevalence of Any RIF Resistance

**Figure 4:**
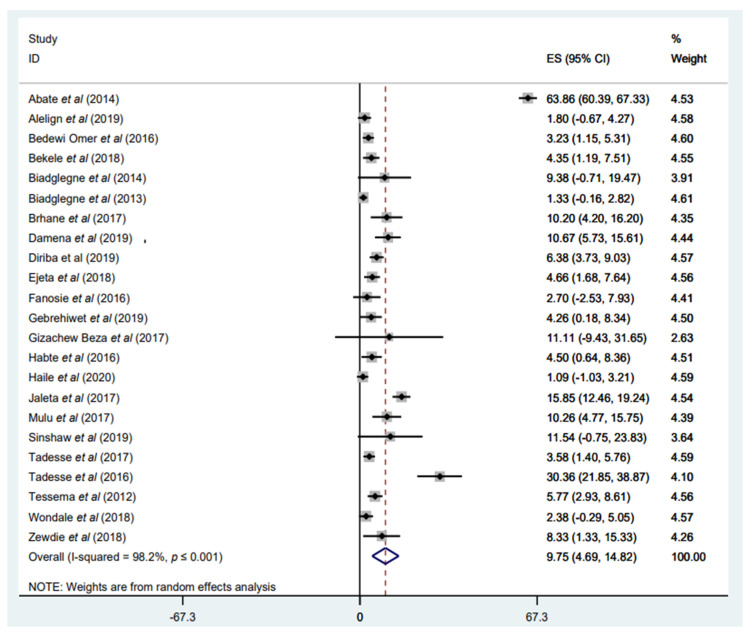
Forest plot showing the weighted pooled prevalence of any rifampicin (RIF) resistance in tuberculosis (TB) patients.

In this meta-analysis, almost all included studies [31,33,34,35,36,37,38,39,40,41,42,43,44,45,46,47,48,49,50,51,52,53,54] reported any-RIF-resistant *M. tuberculosis* isolates (Table 1). The weighted pooled prevalence of any RIF resistance was 9.75% (95% CI: 4.69–14.82%; I^2^ = 98.2%) among a total of 4086 *M. tuberculosis* isolates with available DST results (Figure 4). The funnel plot, as well as Egger’s test result, which indicates a publication bias, and the sensitivity analysis of the included papers, are all presented in a supplementary file (Supplementary Figures S3A–D). Geographically, the prevalence of any RIF resistance varied. Two studies [35,54] reported the highest prevalence of any RIF resistance. The highest incidence of any RIF resistance, with 65.35%, was reported by Abate et al. [54] in central Ethiopia, Addis Ababa. This might be because the study only included retreated patients with TB, and it is anticipated that the incidence of DR-TB is high among patients previously treated with TB [54]. The lowest rate of any-RIF resistance, with 1.09%, was reported by Haile et al. [40] in the Aris zone, Oromia Region (Figure 4).

#### 3.3.4. Prevalence of INH Mono-Resistance

**Figure 5:**
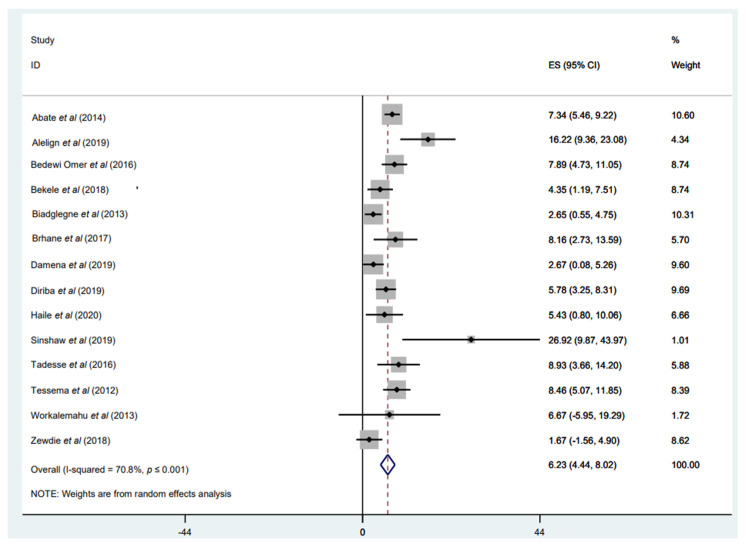
Forest plot showing the weighted pooled prevalence of isoniazid (INH)-monoresistance in tuberculosis (TB) patients.

Mono-resistance to INH was reported in 14 studies [31,32,34,35,37,40,46,47,48,49,51,52,53,54], with a weighted pooled prevalence of 6.23% (95% CI: 4.44–8.02%; I^2^ = 70.8%) among a total of 2655 *M. tuberculosis* isolates (Figure 5). The funnel plot, as well as Egger’s test result, which indicates a publication bias, and the sensitivity analysis of the included papers, are all presented in a supplementary file (Supplementary Figures S4A–D). Geographically, the highest proportion of monoresistance to INH, with 26.92%, was reported by Sinshaw et al. in central Ethiopia, Addis Ababa [37], while the lowest prevalence was 1.67% [31] (Figure 5).

#### 3.3.5. Prevalence of RIF Mono-Resistance

**Figure 6:**
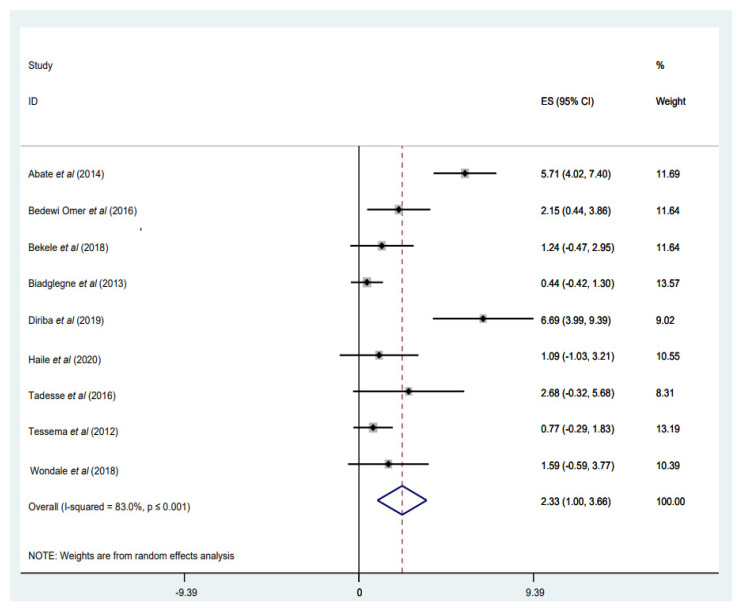
Forest plot showing the weighted pooled prevalence of rifampicin (RIF)-monoresistance in tuberculosis (TB) patients.

Mono-resistance to RIF was reported in nine studies [33,34,35,40,46,49,51,52,54], with a weighted pooled prevalence of 2.33% (95% CI: 1.00–3.66%; I^2^ = 83.0%) among a total of 2781 *M. tuberculosis* isolates (Figure 6). The funnel plot, as well as Egger’s test result, which indicates a publication bias, and the sensitivity analysis of the included papers, are all presented in a supplementary file (Supplementary Figures S5A–D). Two studies have reported the highest proportion of RIF monoresistance at 5.7% [54] and 6.7% [46] (Figure 6).

#### 3.3.6. Prevalence of MDR-TB among Newly Diagnosed Cases

**Figure 7:**
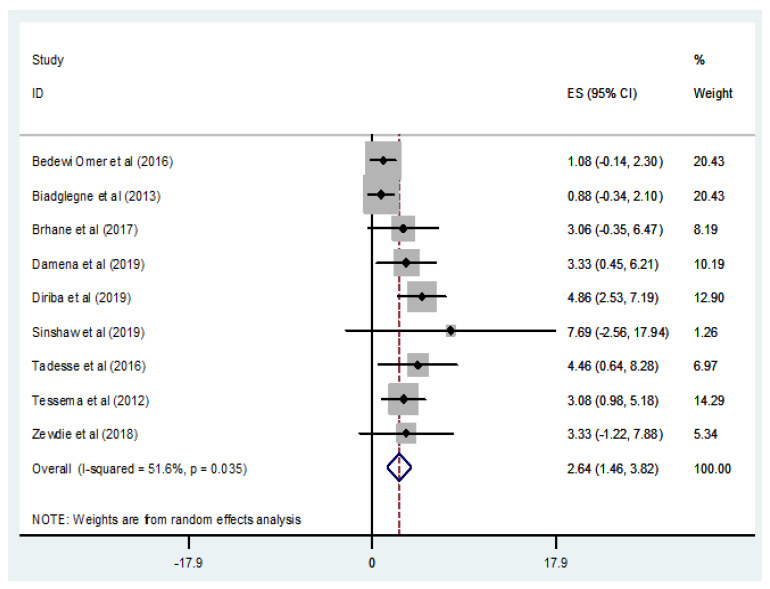
Forest plot showing the weighted pooled prevalence of multidrug-resistant tuberculosis (MDR-TB) in newly diagnosed TB cases.

Out of 14 studies [31,32,33,34,35,37,40,46,47,48,49,52,53,54] that performed DST and evaluated the occurrence of MDR-TB in newly diagnosed TB cases, nine studies [31,34,35,37,46,47,48,49,52] with a total of 1540 *M. tuberculosis* isolates with available DST results reported MDR-TB (*n* = 46) among newly diagnosed TB cases (Table 1). The prevalence of MDR-TB in newly diagnosed cases ranged from 0.88% [49] to 7.69% [37], with a weighted pooled prevalence of 2.64% (95% CI: 1.46–3.82%; I^2^ = 51.6%; *p* = 0.035) (Figure 7). The funnel plot, as well as Egger’s test result, which indicates a publication bias, and the sensitivity analysis of the included papers, are all presented in a supplementary file (Supplementary Figures S6A–E). Geographically, the highest proportion of MDR-TB in newly diagnosed TB cases, with a rate of 7.69%, was reported in central Ethiopia, Addis Ababa [37], while the lowest prevalence, with a rate of 0.88%, was reported in the Amhara region [49] (Figure 7).

#### 3.3.7. Prevalence of MDR-TB among Retreated Patients with TB

**Figure 8:**
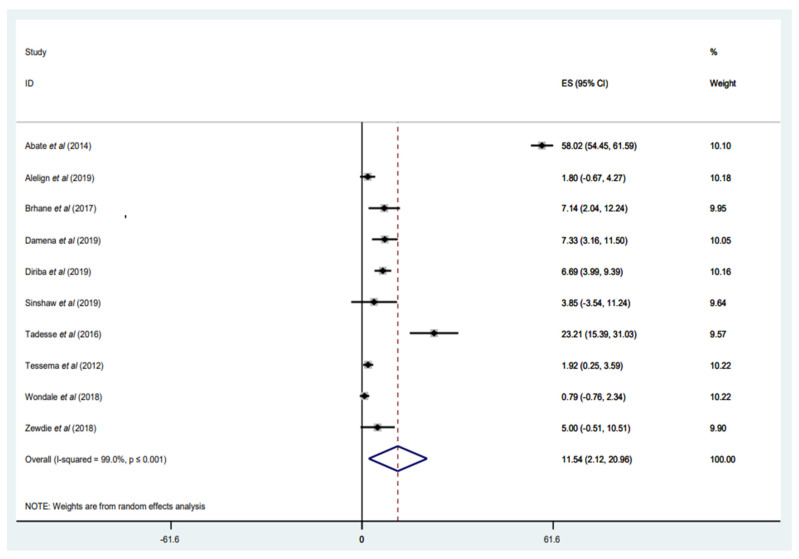
Forest plot showing the weighted pooled prevalence of multidrug-resistant tuberculosis (MDR-TB) among patients retreated with TB.

Fourteen studies [31,32,33,34,35,37,40,46,47,48,49,52,53,54] with a total of 2620 *M. tuberculosis* isolates performed DST and evaluated the occurrence of MDR-TB among retreated patients with TB (Table 1). However, only 10 studies [31,33,34,35,37,46,47,48,53,54] comprising 2008 *M. tuberculosis* isolates have reported the occurrence of MDR-TB (*n* = 505) in retreated patients with TB, with a weighted pooled prevalence of 11.54% (95% CI: 2.12–20.96%; I^2^ = 99.0) (Figure 8). The funnel plot, as well as Egger’s test result, which indicates a publication bias, and the sensitivity analysis of the included papers, are all presented in a supplementary file (Supplementary Figures S7A–D). Geographically, the prevalence of MDR-TB in retreated patients with TB varies widely, ranging from 0.88% [33] to 58.02% [54]. The highest proportion was reported in five studies, ranging from 6.69% to 58.02% [35,46,47,48,54], two of which were conducted in central Ethiopia, Addis Ababa [47,54]. Central Ethiopia had the highest incidence of MDR-TB in retreated patients with TB, with a rate of 58.02% [54], while the lowest prevalence, with a rate of 0.79%, was reported in the Southern Nations, Nationalities, and Peoples’ region [33]. That the highest proportion (58.02%) of MDR-TB in retreated patients with TB was observed in central Ethiopia (Addis Ababa) may be because the study only included retreated patients with TB [54], which increases the likelihood of *M. tuberculosis* strains developing MDR during therapy (Figure 8).

#### 3.3.8. Prevalence of MDR-TB among Overall Patients with TB

**Figure 9:**
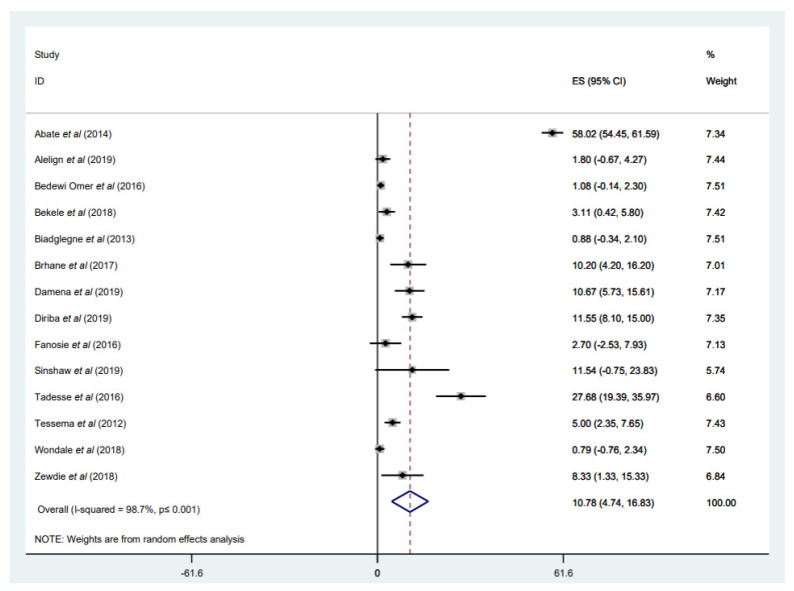
Forest plot showing the weighted pooled prevalence of multidrug-resistant tuberculosis (MDR-TB) among overall TB cases.

Sixteen studies [31,32,33,34,35,37,40,44,46,47,48,49,51,52,53,54] with a total of 2818 *M. tuberculosis* isolates used molecular DST and evaluated the incidence of MDR-TB in all types of TB cases (newly diagnosed and retreated patients with TB) (Table 1). However, only 14 studies [31,33,34,35,37,44,46,47,48,49,51,52,53,54] with a total of 2711 *M. tuberculosis* isolates had reported the occurrence of MDR-TB (*n* = 557) among overall TB cases, with a weighted pooled prevalence of 10.78% (95% CI: 4.74–16.83%; I^2^ = 98.7%) (Figure 9). The funnel plot, as well as Egger’s test result, which indicates a publication bias, and the sensitivity analysis of the included papers, are all presented in a supplementary file (Supplementary Figures S8A–D). The prevalence of MDR-TB among all TB cases differs geographically. Seven studies showed a high proportion of MDR-TB, ranging from 8.33% to 58.02% [31,35,37,46,47,48,54]; four of the studies were conducted in central Ethiopia, Addis Ababa [31,37,47,54]. The highest prevalence of MDR-TB patients (58.02%) among all TB cases was recorded in Addis Ababa [54], which might be because the study included only retreated patients with TB who had previously received anti-TB treatment, and this might increase the likelihood of *M. tuberculosis* strains to develop MDR during therapy. On the other hand, the lowest proportion was observed in the Southern region of Ethiopia [33] and the Amhara region [49] at 0.78% and 0.88%, respectively (Figure 9).

## 4. Discussion

The increasing incidence of DR-TB strain is one of the most serious threats to global TB control efforts [1,15], particularly in resource-constrained countries, where innovative molecular diagnostic technologies and well-equipped laboratory settings are lacking [3,6,7]. In particular, the emergency and spread of MDR/XDR-TB strains in both developing and developed countries need substantial global efforts, resources, and breakthrough diagnostic technology to halt transmission and control the disease [1,55]. Ethiopia, like other LMICs, is experiencing an increase in DR-TB, posing challenges for its public healthcare system and national TB-control efforts [3]. Ethiopia is one of the top 30 countries with the highest rates of TB, DR-TB, and TB/HIV co-infection [3], and MDR-TB was found in 0.71% of newly diagnosed cases and 12.0% of patients previously treated with TB in 2019 [1]. To identify the drug-resistance pattern of *M. tuberculosis* isolates and to ensure effective therapy for patients with TB, all TB-positive individuals must undergo DST for first-and second-line anti-TB drugs [1]. Monitoring drug resistance is critical for developing TB control policies and strategies, as treating drug resistance requires the use of more complicated, expensive, toxic, and less effective second-line antibiotics [1].

In this review, we estimated the weighted pooled prevalence of any anti-TB drug resistance, any INH and RIF resistance, monoresistance to INH and RIF, and MDR-TB in newly diagnosed cases and retreated patients with TB in Ethiopia using molecular DST results from 24 studies included. The pooled prevalence of any anti-TB drug resistance (14.25%) found in our review was lower than Benin’s national survey report, which found a rate of 54% [56], as well as the pooled estimated rates in China (20.1%) [30], and Burundi (16.1%) [57]. However, it was higher than the national survey results in Mozambique (11.4%) [58], Rwanda (12.0%) [59], and the weighted pooled prevalence estimate in Nigeria (12.0%) [6]. This discrepancy may be due to the types of studies used. Our review included only studies that used molecular DST methods, whereas Benin and Rwanda’s national surveys included phenotypic DST results. The other possible reason could be due to study conditions, especially laboratory setups, the sensitivity of drug-resistance diagnostic methods, and the skill of laboratory staff.

INH is a selective prodrug used to treat active TB in the first stage of the disease, and it is commonly given together with RIF, pyrazinamide, and either streptomycin or ethambutol [54]. INH resistance must be assessed regularly because it lowers the chances of TB-treatment success, raises the risk of developing MDR-TB, and decreases the efficacy of INH preventive therapy [60]. The weighted pooled prevalence of any INH resistance found in our review (15.62%) was lower than the 27.9% reported in a national survey in Benin [56], but higher than the 7.9% reported in a national survey in Mozambique [58], Burundi (6.3%) [57], and the pooled prevalence estimated in China (12.0%) [30]. In agreement with our result, one previous study conducted using WHO data (1994–2009) estimated the INH resistance burden and found that INH resistance was considerably higher in the Eastern European region than in any other WHO region, at 44.9%; of those, 33.5% were among new cases and 61.4% were among patients retreated with TB [60]. According to the same report, INH resistance in Africa was found to be 6.3% among newly diagnosed cases and 20% among retreated patients with TB [60].

Despite its prevalence, INH monoresistance has gained less attention than RIF resistance until recently, since INH resistance is more difficult to diagnose using molecular testing and the clinical consequences of INH resistance are unknown [61]. A DST for INH-monoresistance could be conducted on patients with TB, as INH-monoresistance increases the chance of a poor treatment outcome and progression to MDR-TB if not addressed properly [60]. INH-monoresistance is the most common form of DR-TB globally, with estimates rising to 7.0% among newly diagnosed TB cases and 8.0% to 11.0% among previously treated TB cases [62]. From 1994 to 2009, monoresistance to INH was estimated to occur in between 6.4% and 33.5% of new TB cases [60]. The pooled prevalence of INH monoresistance in our review (6.23%) was lower than the 9.3% reported in Benin [56], and Nigeria (11.0%) [6]. However, it was higher than the 1.1% reported in Rwanda [59], and 2.3% in Mozambique [58]. Notably, our review demonstrated that INH-monoresistant TB is prevalent in Ethiopia. The high pooled prevalence of INH monoresistance observed in this review may be due to the use of INH as empiric treatment for HIV-positive and TB-exposed individuals in Ethiopia. Also, further study is required to better comprehend the prevalence and phenotypic and molecular resistance features of INH-monoresistant *M.tuberculosis* strains in Ethiopia.

RIF is a highly effective sterilizing agent against resistant *M. tuberculosis* isolates. Resistance to RIF is most commonly conferred by mutations in the *rpo*B gene, which codes for the RNA polymerase ß-subunit [63]. Besides direct transmission, RIF resistance is acquired by the selection of mutant strains during anti-TB treatment, which is usually the result of improper medication, stock-outs, poor adherence, and/or drug penetration [63]. RIF resistance is the basis of both MDR and XDR-TB, and the key indicator for MDR-TB, and makes patient treatment more difficult [64,65]. In our review, we found that the weighted pooled prevalence of any RIF resistance was 9.75%, which was lower than the reported rate in Benin [56]. Similarly, our result was slightly lower than the prevalence of any RIF-resistant *M. tuberculosis* reported in a meta-analysis study (11.0%) [66]. According to the same study, the African region had a 3.0% prevalence of RIF resistance, while the Western Pacific, European, South-East Asian, and American regions had 23.0%, 10.0%, 6.0%, and 1.0%, respectively [66]. In contrast, our finding was higher than the report in Rwanda, where the rate was 4.0%, with 3.4% in new cases and 19.6% in previously treated patients with TB [59].

Currently, RIF-resistant TB is incorrectly classified as MDR-TB; however, this method could lead to incredibly long and toxic anti-TB drug treatment regimens for patients with TB with RIF-monoresistant TB strains [67]. The availability of diagnostic tests that can detect RIF resistance quickly has increased awareness of the presence of patients with RIF-monoresistant TB, which was previously thought to be uncommon [67]. In our study, we found that the pooled prevalence of RIF monoresistance was 2.33%, which was comparable to the rate of RIF monoresistance reported in Benin (2.2%) [56]. According to Weldegebreal and colleagues, Ethiopia has a prevalence of RIF monoresistance ranging from 0.0% to 2.2% [27]. In contrast, our review result was higher than those reported in other African countries, e.g., Rwanda (0.1%) [59] and Mozambique (0.4%) [58]. Interestingly, our study highlighted that RIF-monoresistance is widespread in Ethiopia, resulting in the misclassification of many patients with RIF-monoresistant TB as MDR-TB patients and their exposure to more toxic, ineffective second-line treatment. Hence, it is crucial to accurately detect RIF-monoresistant TB strains using proper diagnostic methods in order to appropriately treat patients with TB and achieve a good treatment outcome.

A more worrisome feature of TB is the spread of MDR-TB strains. MDR-TB is a form of TB that is difficult and expensive to treat since it is resistant to two important first-line antibiotics, RIF and INH [1]. MDR-TB is an emerging threat that has always played a significant role in the prevention and control of infectious diseases [1]. In our study, we found that MDR-TB had a weighted pooled prevalence of 2.64% among newly diagnosed cases. Our result was consistent with recent reports in Ethiopia by Girum et al. [24] and Eshetie et al. [25], who reported 2.2% and 2.0%, respectively. Similarly, according to the national report, MDR-TB was detected in 2.7% of newly diagnosed patients in Ethiopia [68]. In agreement with this, the first Ethiopian national TB survey found that MDR-TB prevalence was 2.7% [69]. However, our result was marginally higher than a meta-analysis finding in Sub-Saharan African countries (1.5%) [26], and the national survey reports of Ethiopia [3] and Benin [56]. Nevertheless, our result was lower than the reports from elsewhere: Mozambique [58], Rwanda [59], and Nigeria [6].

On the other hand, the weighted pooled prevalence of MDR-TB among previously treated patients with TB (11.54%) in our review was lower than the reports by Eshetie et al. (15.0%) [25] and Girum et al. (21.1%) [24], as well as the WHO (2019) national survey report estimated for Ethiopia (16%) [3]. Similarly, it was lower than a Nigerian meta-analysis study, which found a pooled prevalence of 19.0% [6]. It was, however, similar with the survey results from Benin (11.1%) [56], Mozambique (11.2%) [58], and Rwanda (9.4%) [59]. While previously treated patients with TB are more likely to contract a new infection and develop resistance by gene mutation while on medication, their risk of developing MDR-TB is higher than that of newly diagnosed patients with TB [1,24,25]. On the other hand, several previous studies have demonstrated that MDR is associated with specific *M. tuberculosis* genotypes that have effectively adapted to a particular geographical area and are capable of spreading to the entire population, like the Beijing genotype [66]. Thus, it is necessary to define the genetic diversity of *M. tuberculosis* in a specific geographical area and to analyze its drug-resistance profile to provide effective TB-control measures.

The results of this review may be hampered by the fact that the study was undertaken in TB treatment facilities, which means that its generalizability for the general population is limited. Because the review was confined to studies published in English and within the given data sources, as well as within a few geographical areas, generalization for the entire country may be challenging. Owing to the facility-based nature of the primary study, presumed TB cases may be included, thereby increasing the prevalent estimate.

In conclusion, DR-TB continues to be a major public health problem in Ethiopia. MDR-TB was found to be marginally lower among retreated cases and in overall TB patients compared to the WHO national report (2020); however, MDR-TB among newly diagnosed TB cases remained high. Besides, this review found that monoresistance to INH and RIF is higher than in earlier studies in Ethiopia. Early TB-case detection, proper treatment of both drug-susceptible and DR-TB, and a strict TB-patient follow-up strategy are all important. Rapid and effective molecular diagnostic methods for both drug-susceptible and DR-TB are also critical for the early detection and proper treatment of patients with TB.

## Data Availability

The datasets analyzed during this review can be accessed from the corresponding author upon reasonable request.

## Supplementary Materials

The following supporting information can be downloaded at: https://www.mdpi.com/article/10.3390/tropicalmed7100300/s1, Figures S1A–E; Figures S2A–D; Figrues S3A–D; Figures S4A–D; Figures S5A–D; Figrues S6A–E; Figures S7A–D; Figures S8A–D. Table S1: PRISMA-2020-checklist; Table S2: Search Strategy Medline/PubMed; Table S3: Quality assessments.

## Abbreviations

DR-TB: drug-resistant tuberculosis; DST: drug susceptibility testing; EMB: ethambutol; FLQ: fluoroquinolone; HIV: human immunodeficiency virus; INH: isoniazid; MDR-TB: multidrug-resistant tuberculosis; PRISMA: Preferred Reporting Items for Systematic Review and Meta-analysis; RIF: rifampicin; TB: tuberculosis; WHO: World Health Organization; XDR-TB: extensively drug-resistant tuberculosis.
