# Supplementary material for: Mycobacterium tuberculosis Drug Resistance in Ethiopia: An Updated Systematic Review and Meta-Analysis
Source: Trop Med Infect Dis. 2022 Oct 14;7(10):300. doi: 10.3390/tropicalmed7100300 (PMC9611116; doi:10.3390/tropicalmed7100300)
Supplement: Supplementary file 1 [file tropicalmed-07-00300-s001.zip › tropicalmed-1879113-figure .pdf]

Supplementary Figures: Funnel plots, and the Egger's test result, which shows a publication bias, and the sensitivity analysis of included papers during this meta-analysis to estimate the weighted pooled prevalence of any anti-TB drug resistance, any-INH and RIF resistance, INH and RIF-monoresistance, and MDR rate among TB patients in Ethiopia.

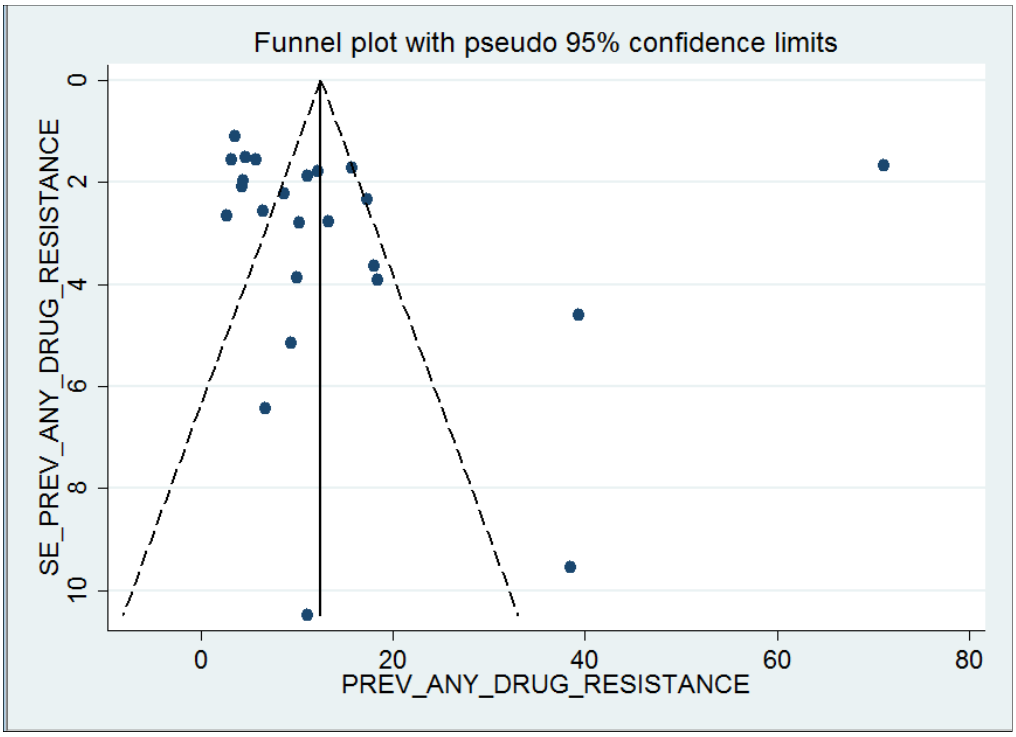

Figure S1A. Funnel plot for publication bias, PREV (prevalence) of any anti-TB resistance represented in the x-axis and SE (standard error) of the prevalence of any anti-TB drug resistance in the y-axis.

```
Tests for Publication Bias

Begg's Test

  adj. Kendall's Score (P-Q) =      104
          Std. Dev. of Score =    40.32
           Number of Studies =       24
                          z  =     2.58
                    Pr > |z| =    0.010
                          z  =     2.55 (continuity corrected)
                    Pr > |z| =    0.011 (continuity corrected)

Egger's test
------------------------------------------------------------------------------
     Std_Eff |      Coef.   Std. Err.      t    P>|t|     [95% Conf. Interval]
-------------+----------------------------------------------------------------
       slope |   9.066489   8.442159     1.07   0.294    -8.441477    26.57445
        bias |   1.754792   3.896781     0.45   0.657    -6.326638    9.836222
------------------------------------------------------------------------------

.
```

Figure S1B. The results of Begg's and Egger's tests for publication bias in estimating the weighted pooled prevalence of any anti-TB drug resistance.

```
Meta-analysis

       |  Pooled      95% CI         Asymptotic      No. of
Method |     Est    Lower   Upper  z_value  p_value   studies
-------+----------------------------------------------------
Fixed  |  12.501   11.634  13.368   28.269    0.000     24
Random |  14.248    7.053  21.443    3.881    0.000

Test for heterogeneity: Q= 1486.500 on 23 degrees of freedom (p= 0.000)
Moment-based estimate of between studies variance = 308.528

Trimming estimator: Linear
Meta-analysis type: Random-effects model

iteration |  estimate    Tn    # to trim     diff
----------+--------------------------------------
    1     |   14.248     91        0          300
    2     |   14.248     91        0            0

Note: no trimming performed; data unchanged

Filled
Meta-analysis

       |  Pooled      95% CI         Asymptotic      No. of
Method |     Est    Lower   Upper  z_value  p_value   studies
-------+----------------------------------------------------
Fixed  |  12.501   11.634  13.368   28.269    0.000     24
Random |  14.248    7.053  21.443    3.881    0.000

Test for heterogeneity: Q= 1486.500 on 23 degrees of freedom (p= 0.000)
Moment-based estimate of between studies variance = 308.528
```

Figure S1C. The trim and fill analysis result for publication bias, in estimating the weighted pooled prevalence of any anti-TB drug resistance.

Study ommited	Coef.	[95% Conf.	Interval]
Abate et al	10.634086	7.9720407	13.296131
Alelign et al	14.0863	6.6789632	21.493637
Bedewi Omer et al	14.399507	6.818614	21.9804
Bekele et al	14.503662	6.9925523	22.014772
Biadglegne et al	14.452383	7.0763793	21.828386
Biadglegne et al	14.641706	7.0067387	22.276674
Brhane et al	14.069606	6.6709189	21.468292
Damena et al	14.294627	6.8358917	21.753363
Diriba et al	14.350941	6.7483902	21.953491
Ejeta et al	14.691198	7.0650153	22.317381
Fanosie et al	14.766131	7.3321872	22.200075
Gebrehiwet et al	14.701543	7.2006269	22.202459
Gizachew Beza et al	14.352984	7.0293779	21.676592
Habte et al	14.693904	7.1746988	22.213108
Haile et al	14.598554	7.1353321	22.061775
Jaleta et al	14.189034	6.5761285	21.801937
Mulu et al	14.427903	6.9720283	21.883778
Sinshaw et al	13.4011	6.0882068	20.713995
Tadesse et al	14.747752	6.9784369	22.517067
Tadesse et al	13.178613	5.8748507	20.482374
Tessema et al	14.117094	6.6266594	21.607529
Wondale et al	14.757228	7.1728797	22.341576
Workalemahu et al	14.551004	7.1934738	21.908535
Zewdie et al	14.434855	7.0303683	21.839342

Figure S1D. Results of a sensitivity analysis assessing the between-study heterogeneity, in estimating the weighted pooled prevalence of any anti-TB drug resistance.

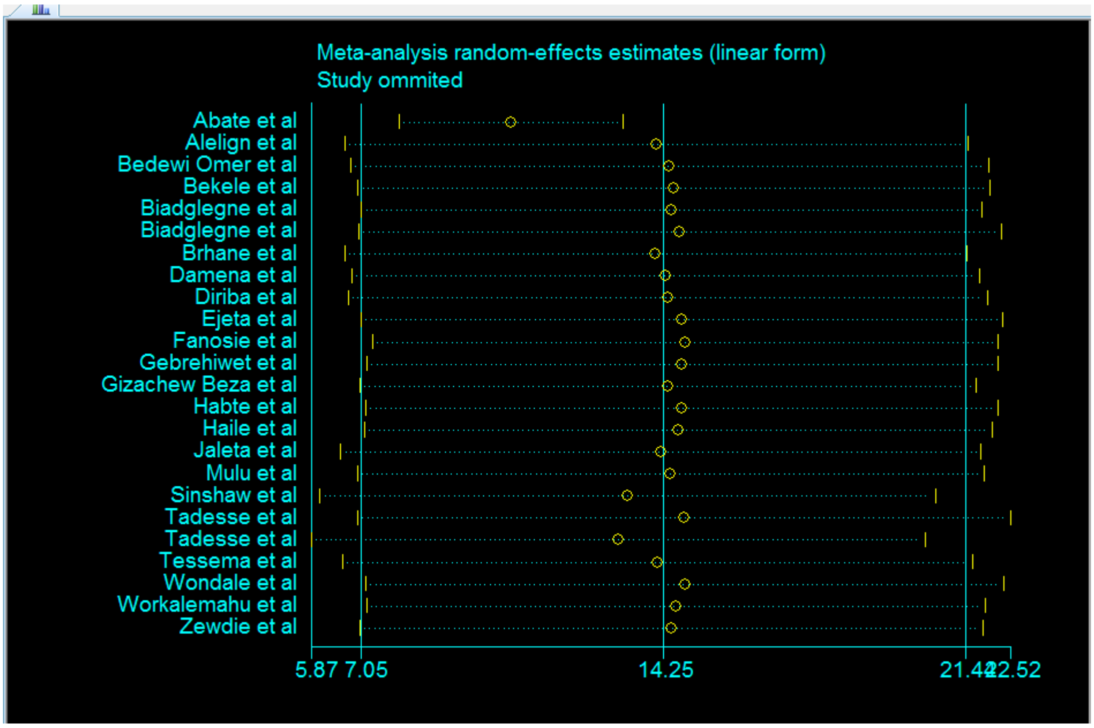

Figure S1E. Results of a sensitivity analysis assessing the between-study heterogeneity in estimating the weighted pooled prevalence of any anti-TB drug resistance.

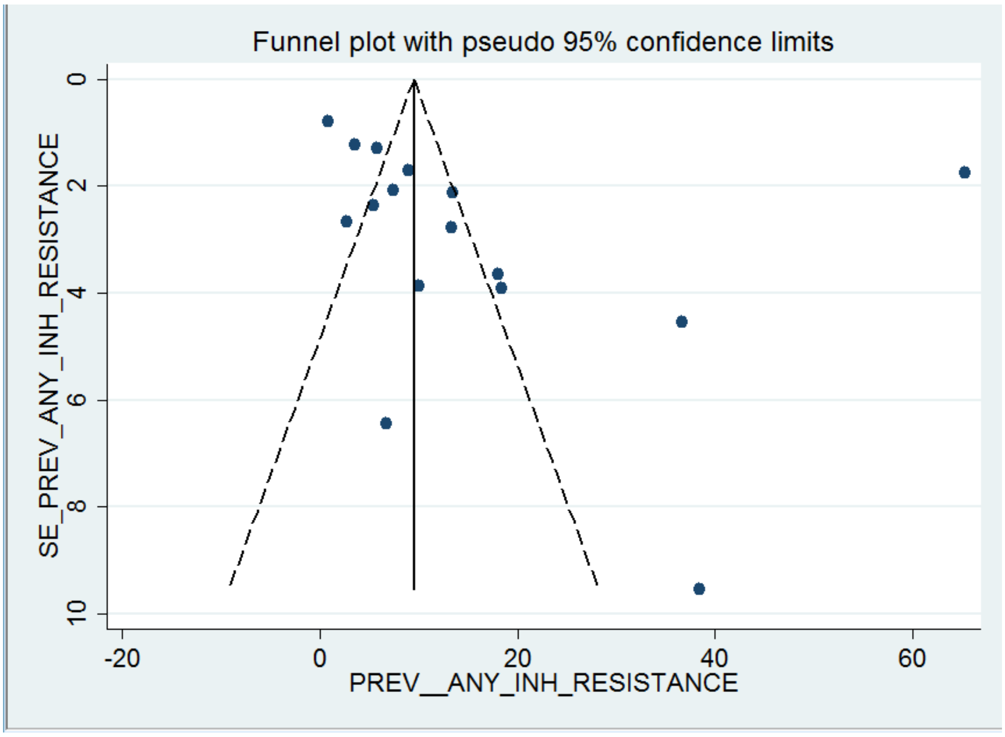

Figure S2A. Funnel plot for publication bias, PREV (prevalence) of any INH resistance represented in the x-axis and SE (standard error) of the prevalence of any INH resistance in the y-axis.

Tests for Publication Bias

Begg's Test

```
adj. Kendall's Score (P-Q) =      56
        Std. Dev. of Score =   22.21
         Number of Studies =      16
                         z =    2.52
                Pr > |z|   =   0.012
                         z =    2.48 (continuity corrected)
                Pr > |z|   =   0.013 (continuity corrected)
```

Egger's test

Std_Eff	Coef.	Std. Err.	t	P>|t|	[95% Conf.	Interval]
slope	.9842021	7.772881	0.13	0.901	-15.68697	17.65537
bias	5.440917	4.225539	1.29	0.219	-3.621963	14.5038

Figure S2B. The results of Begg's and Egger's tests for publication bias, in estimating the weighted pooled prevalence of any INH resistance.

Study ommited	Coef.	[95% Conf.	Interval]
Abate et al	10.766405	7.251575	14.281235
Alelign et al	15.465397	6.2448821	24.685913
Bedewi Omer et al	16.105616	6.4984732	25.712759
Bekele et al	16.199331	6.7429733	25.655689
Biadglegne et al	16.497324	6.5698962	26.424751
Brhane et al	15.442509	6.2353711	24.649647
Damena et al	15.786048	6.4752922	25.096804
Diriba et al	16.342102	6.4232349	26.260971
Fanosie et al	16.509405	7.2008076	25.818003
Haile et al	16.330784	6.955256	25.706314
Sinshaw et al	14.405824	5.3408899	23.470758
Tadesse et al	14.250112	5.1818862	23.318336
Tessema et al	15.78552	6.3537946	25.217245
Wondale et al	16.695992	6.6193614	26.772621
Workalemahu et al	16.169519	7.0207782	25.318262
Zewdie et al	15.998906	6.7708254	25.226986
Combined	15.620452	6.7724494	24.468454

Figure S2C. Results of a sensitivity analysis assessing the between-study heterogeneity in estimating the weighted pooled prevalence of any INH resistance.

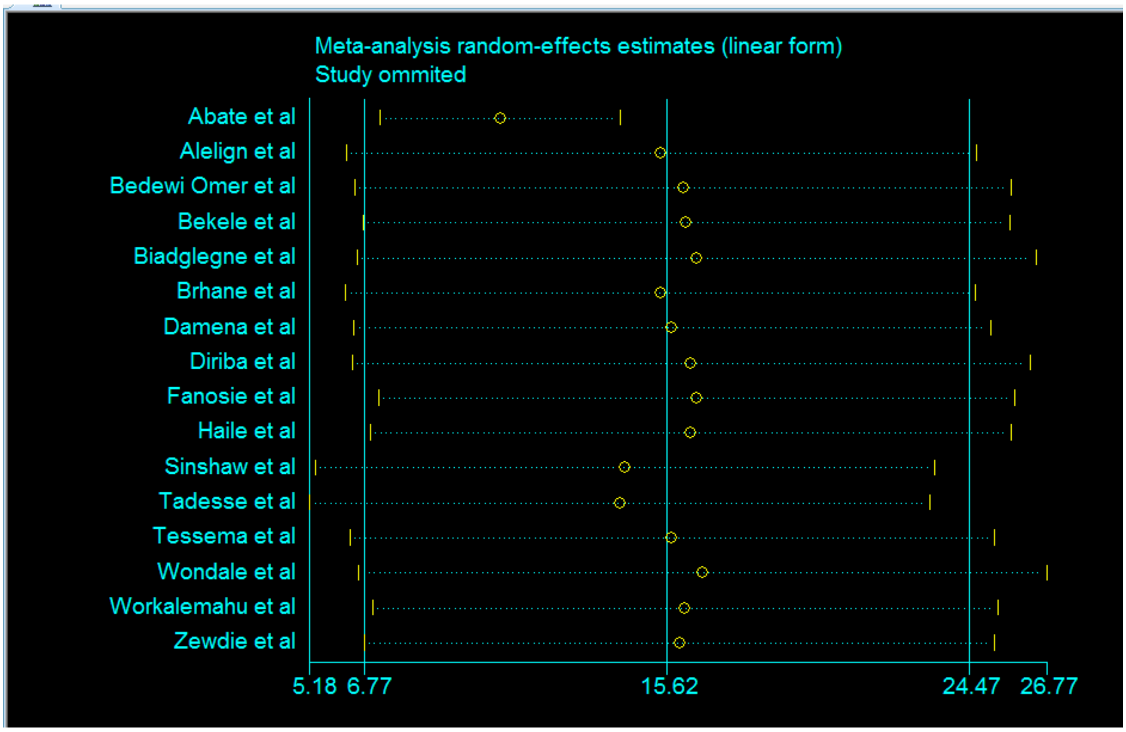

Figure S2D. Results of a sensitivity analysis assessing the between-study heterogeneity in estimating the weighted pooled prevalence of any INH resistance.

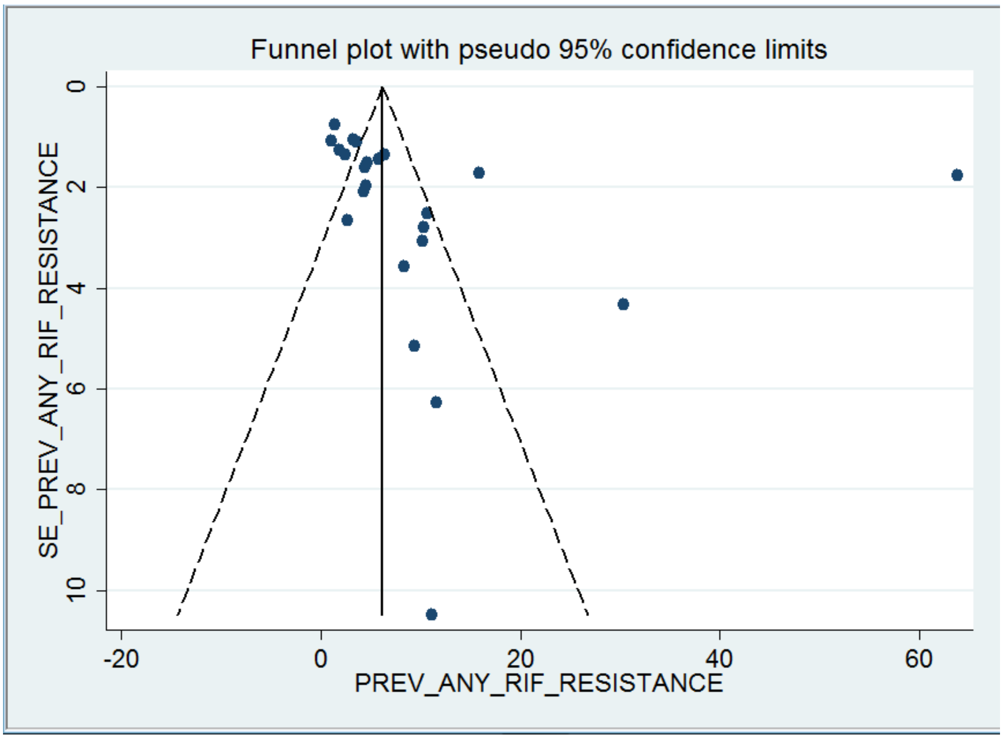

Figure S3A. Funnel plot for publication bias, PREV (prevalence) of any RIF resistance represented in the x-axis and SE (standard error) of the prevalence of any RIF resistance in the y-axis.

```
Tests for Publication Bias

Begg's Test

  adj. Kendall's Score (P-Q) =      121
          Std. Dev. of Score =    37.86
           Number of Studies =       23
                           z =     3.20
                    Pr > |z| =    0.001
                           z =     3.17 (continuity corrected)
                    Pr > |z| =    0.002 (continuity corrected)

Egger's test
```

Std_Eff	Coef.	Std. Err.	t	P>|t|	[95% Conf.	Interval]
slope	-.3606184	5.206953	-0.07	0.945	-11.18907	10.46783
bias	4.6605	3.289899	1.42	0.171	-2.18122	11.50222

Figure S3B. The results of Begg's and Egger's tests for publication bias, in estimating the weighted pooled prevalence of any RIF resistance.

Study	ES	[95% Conf.	Interval]	% Weight
Abate et al (2014)	63.860	60.391	67.329	4.53
Alelign et al (2019)	1.800	-0.670	4.270	4.58
Bedewi Omer et al (2	3.230	1.152	5.308	4.60
Bekele et al (2018)	4.350	1.194	7.506	4.55
Biadglegne et al (20	9.380	-0.714	19.474	3.91
Biadglegne et al (20	1.330	-0.160	2.820	4.61
Brhane et al (2017)	10.200	4.203	16.197	4.35
Damena et al (2019)	10.670	5.731	15.609	4.44
Diriba et al (2019)	6.380	3.734	9.026	4.57
Ejeta et al (2018)	4.660	1.681	7.639	4.56
Fanosie et al (2016)	2.700	-2.533	7.933	4.41
Gebrehiwet et al (20	4.260	0.183	8.337	4.50
Gizachew Beza et al	11.110	-9.430	31.650	2.63
Habte et al (2016)	4.500	0.639	8.361	4.51
Haile et al (2020)	1.090	-1.027	3.207	4.59
Jaleta et al (2017)	15.850	12.459	19.241	4.54
Mulu et al (2017)	10.260	4.772	15.748	4.39
Sinshaw et al (2019)	11.540	-0.749	23.829	3.64
Tadesse et al (2017)	3.580	1.404	5.756	4.59
Tadesse et al (2016)	30.360	21.854	38.866	4.10
Tessema et al (2012)	5.770	2.928	8.612	4.56
Wondale et al (2018)	2.380	-0.286	5.046	4.57
Zewdie et al (2018)	8.330	1.333	15.327	4.26
D+L pooled ES	9.754	4.687	14.821	100.00

Figure S3C. Results of a sensitivity analysis assessing the between-study heterogeneity in estimating the weighted pooled prevalence of any RIF resistance.

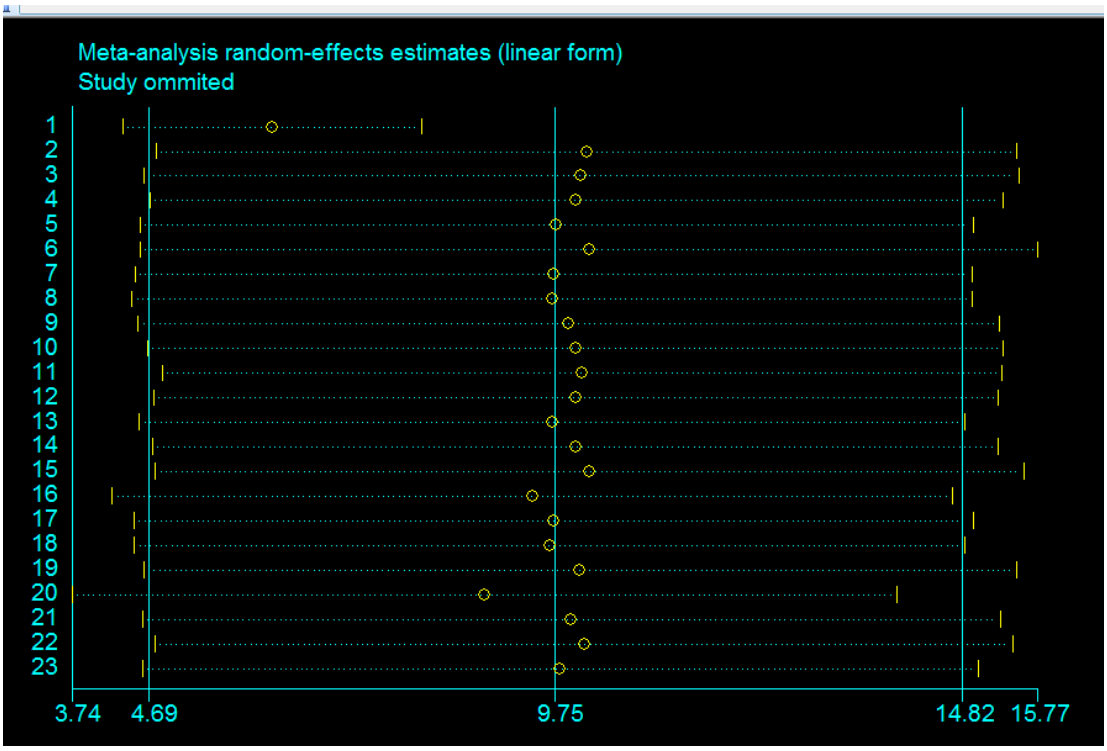

Figure S3D. Results of a sensitivity analysis assessing the between-study heterogeneity in estimating the weighted pooled prevalence of any RIF resistance.

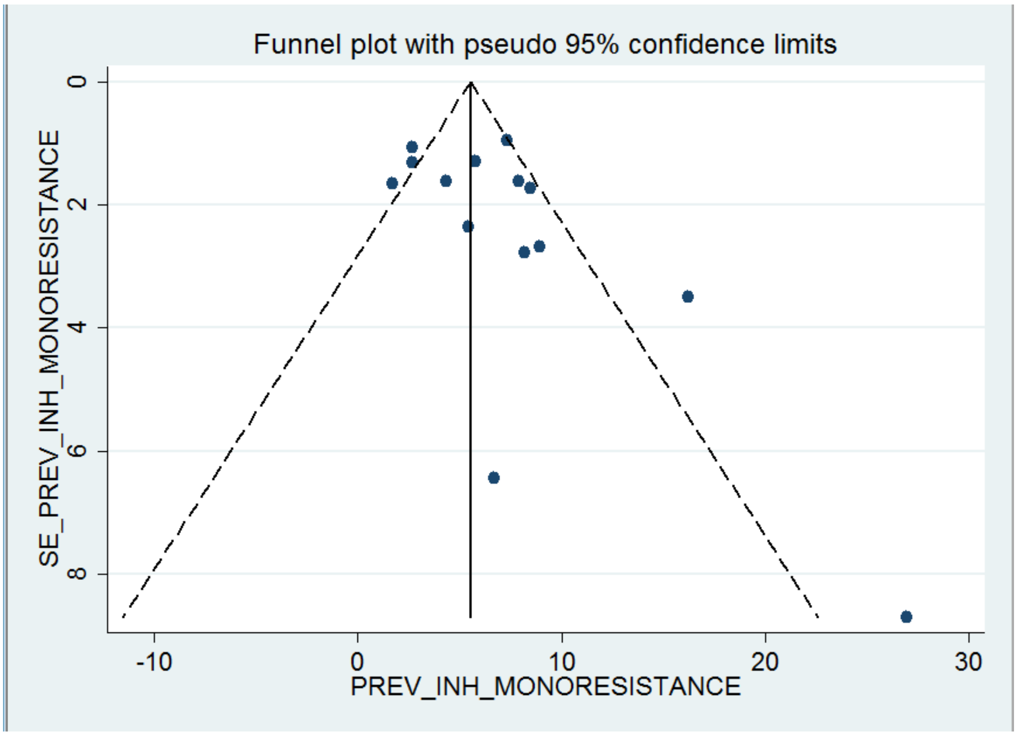

Figure S4A. Funnel plot for publication bias, PREV (prevalence) of INH-mono-resistance represented in the x-axis and SE (standard error) of the prevalence of INH-monoresistance in the y-axis.

```
Tests for Publication Bias

Begg's Test

  adj. Kendall's Score (P-Q) =        26
          Std. Dev. of Score =     18.24 (corrected for ties)
           Number of Studies =        14
                           z =      1.43
                    Pr > |z| =     0.154
                           z =      1.37 (continuity corrected)
                    Pr > |z| =     0.170 (continuity corrected)

Egger's test
```

Std_Eff	Coef.	Std. Err.	t	P>|t|	[95% Conf.	Interval]
slope	2.644842	1.693552	1.56	0.144	-1.045092	6.334776
bias	1.945293	1.02949	1.89	0.083	-.2977735	4.18836

Figure S4B. The results of Begg's and Egger's tests for publication bias, in estimating the weighted pooled prevalence of INH-mono-resistance.

Study ommited	Coef.	[95% Conf.	Interval]
Abate et al	6.1597729	4.1907721	8.1287737
Alelign et al	5.7031174	4.0515203	7.3547144
Bedewi Omer et al	6.0966754	4.1926084	8.000742
Bekele et al	6.4611177	4.5183749	8.4038601
Biadglegne et al	6.6141939	4.7760353	8.4523525
Brhane et al	6.1300974	4.2667284	7.9934669
Damena et al	6.6073847	4.7325659	8.4822035
Diriba et al	6.3603587	4.3626413	8.3580761
Haile et al	6.3239965	4.4235148	8.2244787
Sinshaw et al	5.9727831	4.2795935	7.6659727
Tadesse et al	6.0693555	4.2182636	7.9204478
Tessema et al	6.0341825	4.1583228	7.9100423
Workalemahu et al	6.2399778	4.4135094	8.0664463
Zewdie et al	6.6285348	4.8085175	8.4485521
Combined	6.2324784	4.4448835	8.0200733

Figure S4C. Results of a sensitivity analysis assessing the between-study heterogeneity in estimating the weighted pooled prevalence of INH mono-resistance.

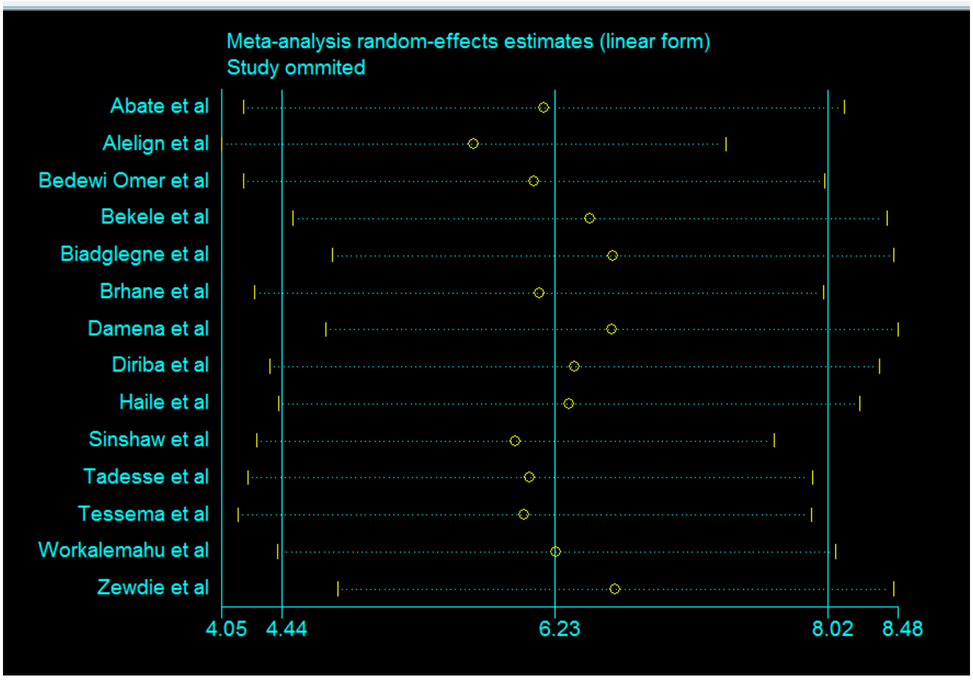

Figure S4D. Results of a sensitivity analysis assessing the between-study heterogeneity in estimating the weighted pooled prevalence of INH mono-resistance.

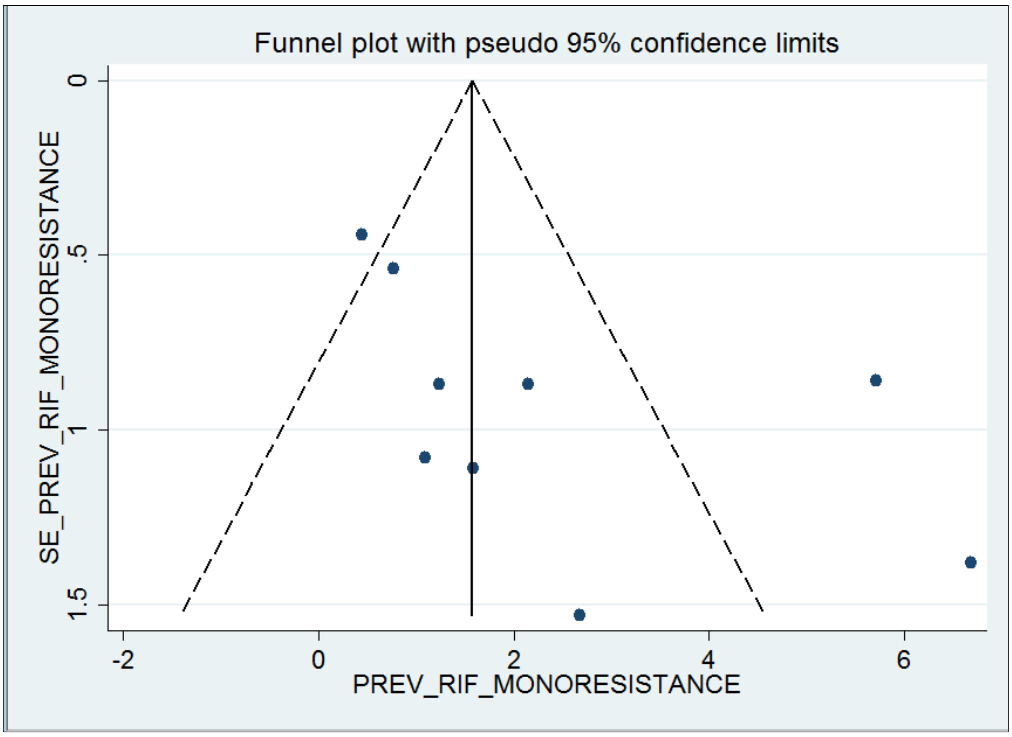

Figure S5A. Funnel plot for publication bias, PREV (prevalence) of RIF-mono-resistance represented in the x-axis and SE (standard error) of the prevalence of any RIF-mono-resistance in the y-axis.

```
Tests for Publication Bias

Begg's Test

  adj. Kendall's Score (P-Q) =      15
          Std. Dev. of Score =    9.54 (corrected for ties)
           Number of Studies =       9
                          z  =    1.57
                    Pr > |z| =   0.116
                          z  =    1.47 (continuity corrected)
                    Pr > |z| =   0.142 (continuity corrected)

Egger's test
```

Std_Eff	Coef.	Std. Err.	t	P>|t|	[95% Conf.	Interval]
slope	-1.051054	1.341061	-0.78	0.459	-4.22216	2.120053
bias	3.705042	1.743399	2.13	0.071	-.4174408	7.827525

Figure S5B. The results of Begg's and Egger's tests for publication bias, in estimating the weighted pooled prevalence of RIF-mono-resistance.

Study ommited	Coef.	[95% Conf.	Interval]
Abate et al	1.7387358	.69534922	2.7821224
Bedewi Omer et al	2.3714638	.87437099	3.8685567
Bekele et al	2.491853	.99005461	3.9936512
Biadglegne et al	2.6374092	1.1291921	4.1456261
Diriba et al	1.8761458	.66537589	3.0869157
Haile et al	2.4900289	1.0243262	3.9557316
Tadesse et al	2.3043659	.88516986	3.723562
Tessema et al	2.591362	1.0064106	4.1763134
Wondale et al	2.4294624	.96440083	3.8945239
Combined	2.3297366	1.0001319	3.6593414

Figure S5C. Results of a sensitivity analysis assessing the between-study heterogeneity in estimating the weighted pooled prevalence of RIF mono-resistance.

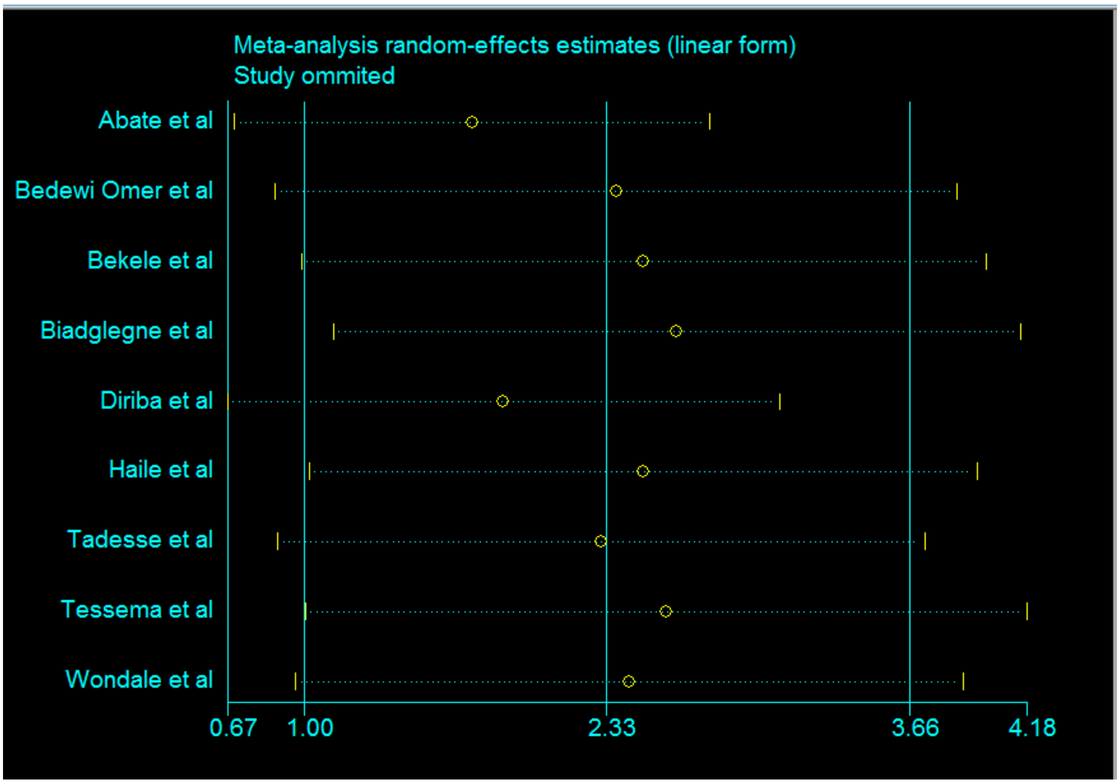

Figure S5D. Results of a sensitivity analysis assessing the between-study heterogeneity in estimating the weighted pooled prevalence of RIF mono-resistance.

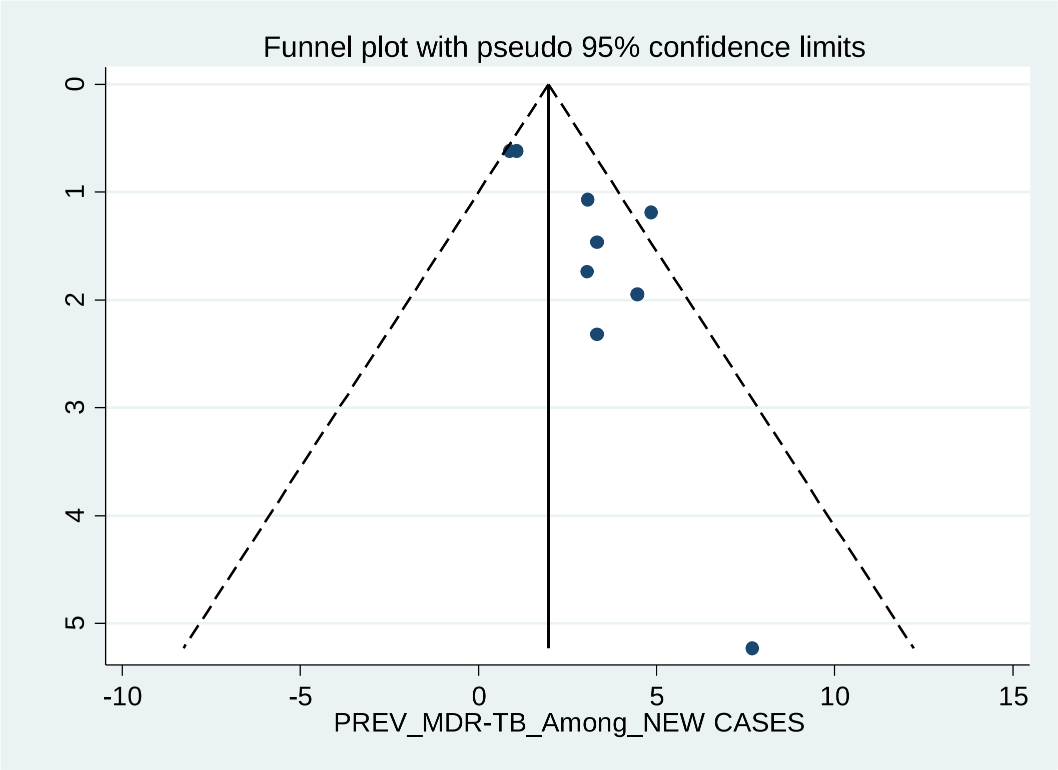

Figure S6A. Funnel plot for publication bias, PREV (prevalence) of MDR-TB among new cases represented in the x-axis, and SE (standard error) of the prevalence of MDR-TB among new cases in the y-axis.

```
Tests for Publication Bias

Begg's Test

  adj. Kendall's Score (P-Q) =        7
          Std. Dev. of Score =     9.54 (corrected for ties)
           Number of Studies =        9
                          z  =     0.73
                  Pr > |z|   =    0.463
                          z  =     0.63 (continuity corrected)
                  Pr > |z|   =    0.529 (continuity corrected)

Egger's test
------------------------------------------------------------------------------
     Std_Eff |      Coef.   Std. Err.      t    P>|t|     [95% Conf. Interval]
-------------+----------------------------------------------------------------
       slope |   -.0185747   .6630288   -0.03   0.978    -1.586389    1.549239
        bias |    2.164214    .627485    3.45   0.011     .6804475     3.64798
------------------------------------------------------------------------------
```

Figure S6B. The results of Begg's and Egger's tests for publication bias, in estimating the weighted pooled prevalence of MDR-TB among new cases.

```
Meta-analysis

       |  Pooled      95% CI         Asymptotic      No. of
Method |     Est   Lower   Upper  z_value  p_value   studies
-------+----------------------------------------------------
Fixed  |   1.966   1.275   2.656    5.581    0.000       9
Random |   2.643   1.465   3.821    4.397    0.000

Test for heterogeneity: Q= 16.544 on 8 degrees of freedom (p= 0.035)
Moment-based estimate of between studies variance =  1.384

Trimming estimator: Linear
Meta-analysis type: Random-effects model

iteration |  estimate    Tn    # to trim     diff
----------+--------------------------------------
     1    |     2.643    34         3          45
     2    |     1.756    42         5          16
     3    |     1.519    42         5           0

Filled
Meta-analysis

       |  Pooled      95% CI         Asymptotic      No. of
Method |     Est   Lower   Upper  z_value  p_value   studies
-------+----------------------------------------------------
Fixed  |   1.423   0.794   2.052    4.435    0.000      14
Random |   1.638   0.477   2.800    2.764    0.006

Test for heterogeneity: Q= 31.745 on 13 degrees of freedom (p= 0.003)
Moment-based estimate of between studies variance =  2.325
```

Figure S6C. The trim and fill analysis result for publication bias, in estimating the weighted pooled prevalence of MDR-TB among new cases.

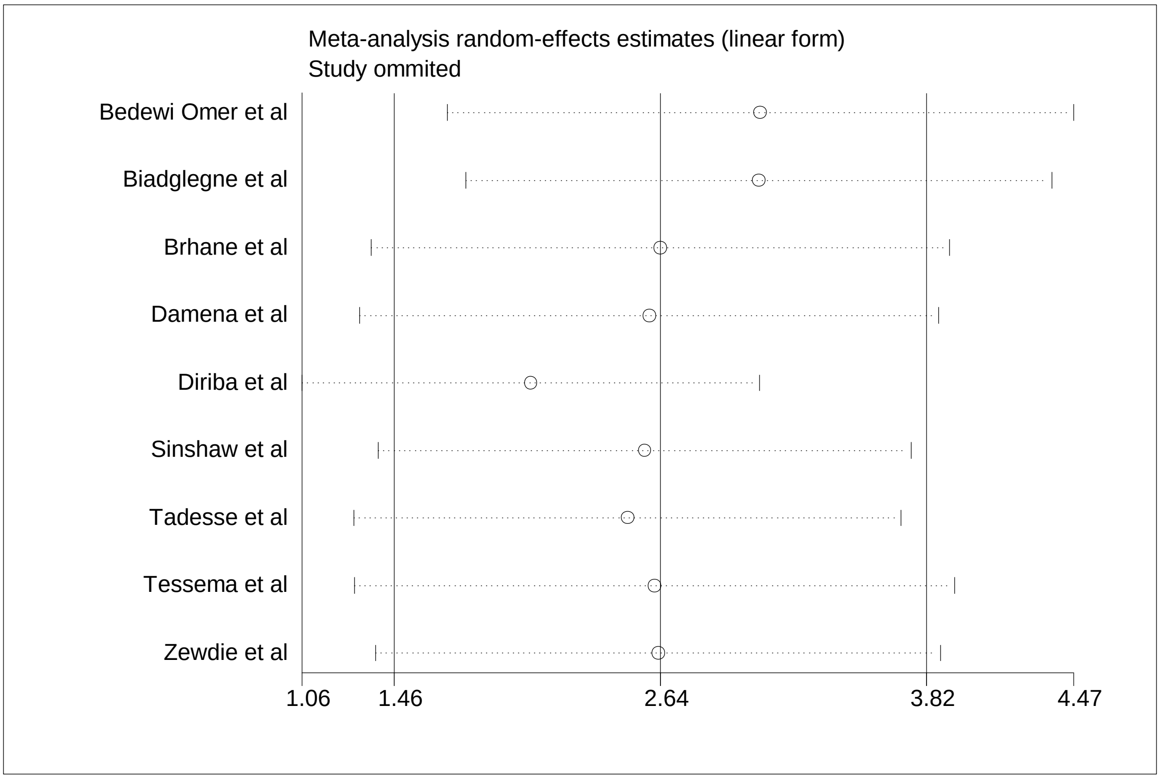

Figure S6D. Results of a sensitivity analysis assessing the between-study heterogeneity in estimating the weighted pooled prevalence of MDR-TB among new cases.

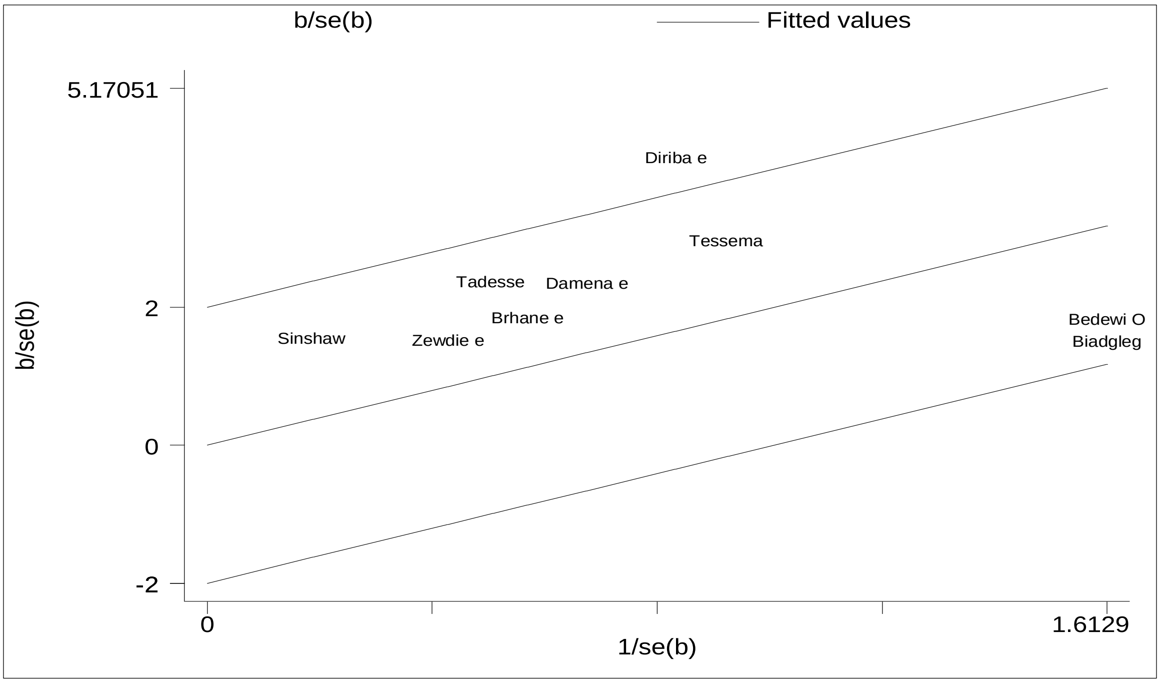

Figure S6E. Results of a sensitivity analysis assessing the between-study heterogeneity in estimating the weighted pooled prevalence of MDR-TB among new cases.

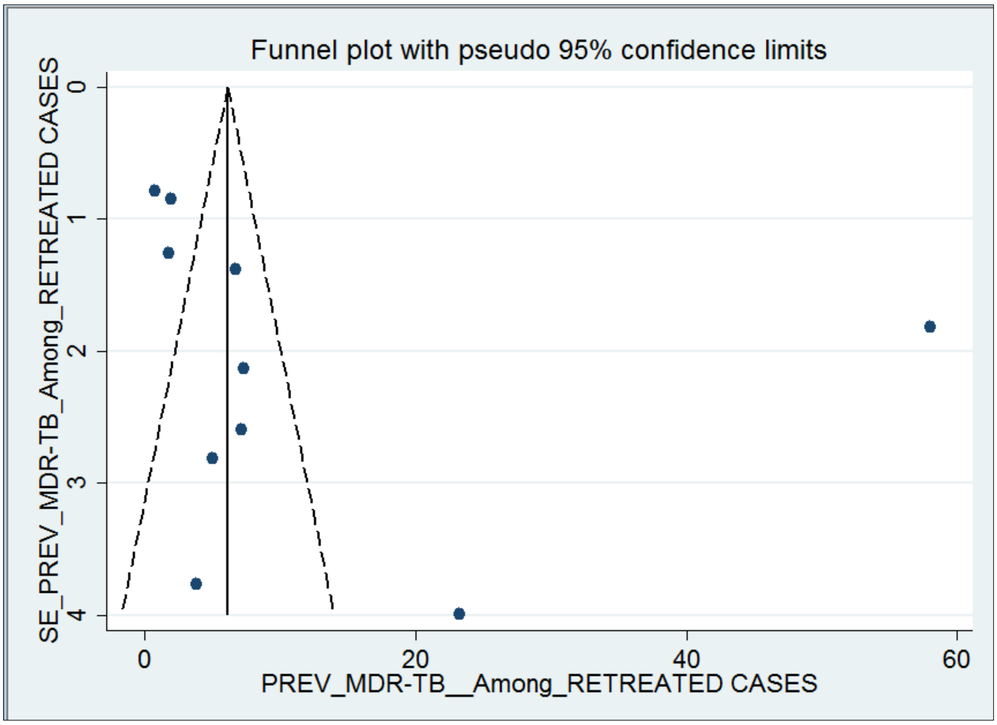

Figure S7A. Funnel plot for publication bias, PREV(prevalence) of MDR-TB among retreated cases represented in the x-axis, and SE (standard error) of the prevalence of MDR-TB among retreated cases in the y-axis.

```
Tests for Publication Bias

Begg's Test

  adj. Kendall's Score (P-Q) =      17
          Std. Dev. of Score =   11.18
           Number of Studies =      10
                          z  =    1.52
                    Pr > |z| =   0.128
                          z  =    1.43 (continuity corrected)
                    Pr > |z| =   0.152 (continuity corrected)

Egger's test
```

Std_Eff	Coef.	Std. Err.	t	P>|t|	[95% Conf.	Interval]
slope	-3.329322	9.056901	-0.37	0.723	-24.21457	17.55593
bias	7.698069	6.449778	1.19	0.267	-7.175145	22.57128

Figure S7B. The results of Begg's and Egger's tests for publication bias, in estimating the weighted pooled prevalence of MDR-TB among retreated cases.

Study ommited	Coef.	[95% Conf.	Interval]
Abate et al	5.2948809	2.6722052	7.9175563
Alelign et al	12.643049	1.8209696	23.465128
Brhane et al	12.024216	1.8749261	22.173506
Damena et al	12.008809	1.7462465	22.271372
Diriba et al	12.089459	1.3518326	22.827087
Sinshaw et al	12.358165	2.3422689	22.37406
Tadesse et al	10.300491	.39830282	20.202681
Tessema et al	12.635528	.90150845	24.369547
Wondale et al	12.763624	1.0636098	24.46364
Zewdie et al	12.256725	2.1419365	22.371513
Combined	11.536382	2.1170587	20.955706

Figure S7C. Results of a sensitivity analysis assessing the between-study heterogeneity in estimating the weighted pooled prevalence of MDR-TB among retreated cases.

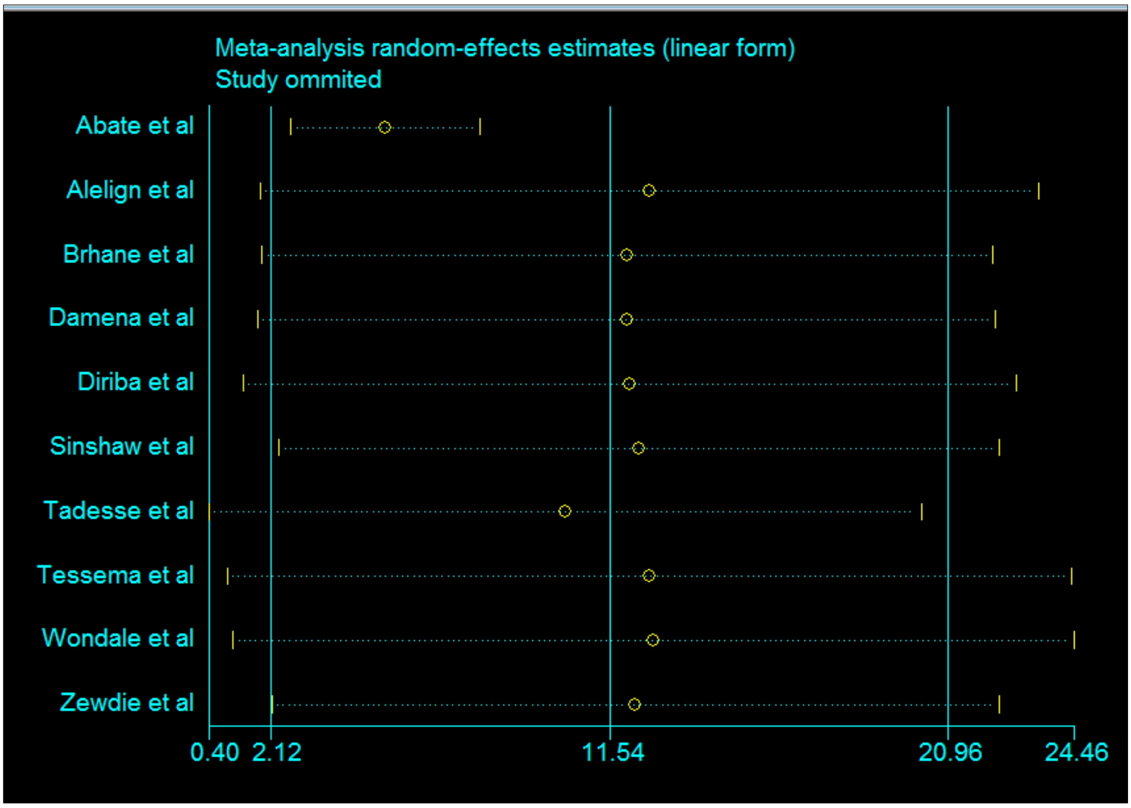

Figure S7D. Results of a sensitivity analysis assessing the between-study heterogeneity in estimating the weighted pooled prevalence of MDR-TB among retreated cases.

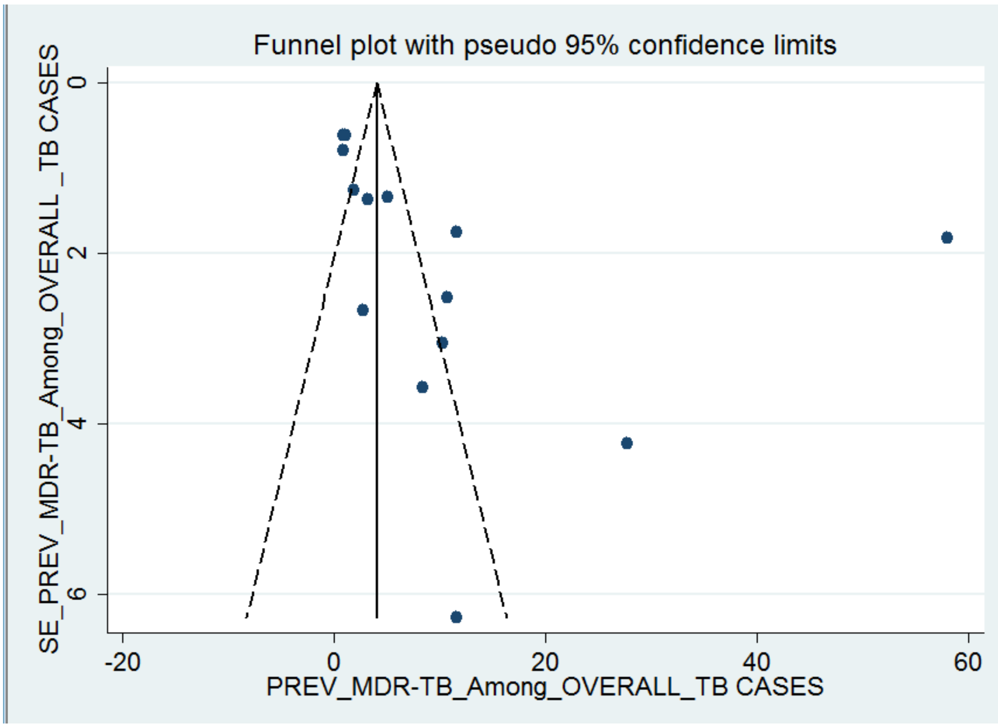

Figure S8A. Funnel plot for publication bias, PREV (prevalence) of MDR-TB among overall TB cases represented in the x-axis, and SE (standard error) of the prevalence of MDR-TB among overall TB cases in the y-axis.

```
Tests for Publication Bias

Begg's Test

  adj. Kendall's Score (P-Q) =      48
          Std. Dev. of Score =   18.24 (corrected for ties)
           Number of Studies =      14
                           z  =    2.63
                    Pr > |z| =   0.008
                           z  =    2.58 (continuity corrected)
                    Pr > |z| =   0.010 (continuity corrected)

Egger's test
```

Std_Eff	Coef.	Std. Err.	t	P>|t|	[95% Conf.	Interval]
slope	-2.934549	4.665809	-0.63	0.541	-13.10047	7.231374
bias	6.972379	3.884858	1.79	0.098	-1.491999	15.43676

Figure S8B. The results of Begg's and Egger's tests for publication bias, in estimating the weighted pooled prevalence of MDR-TB among overall TB cases.

Study ommited	Coef.	[95% Conf.	Interval]
Abate et al	5.6974387	3.4588323	7.9360447
Alelign et al	11.517895	4.9541521	18.081636
Bedewi Omer et al	11.607622	4.3049107	18.910334
Bekele et al	11.409986	4.8829365	17.937037
Biadglegne et al	11.623203	4.3359981	18.910408
Brhane et al	10.829391	4.5218172	17.136963
Damena et al	10.794916	4.4681644	17.121668
Diriba et al	10.727485	4.3564563	17.098513
Fanosie et al	11.407612	5.0691738	17.74605
Sinshaw et al	10.737627	4.5007482	16.974506
Tadesse et al	9.587923	3.4001679	15.775679
Tessema et al	11.259382	4.7253251	17.79344
Wondale et al	11.618974	4.6620841	18.575863
Zewdie et al	10.965412	4.6682591	17.262566
Combined	10.783221	4.7379156	16.828526

Figure S8C. Results of a sensitivity analysis assessing the between-study heterogeneity in estimating the weighted pooled prevalence of MDR-TB among overall TB cases.

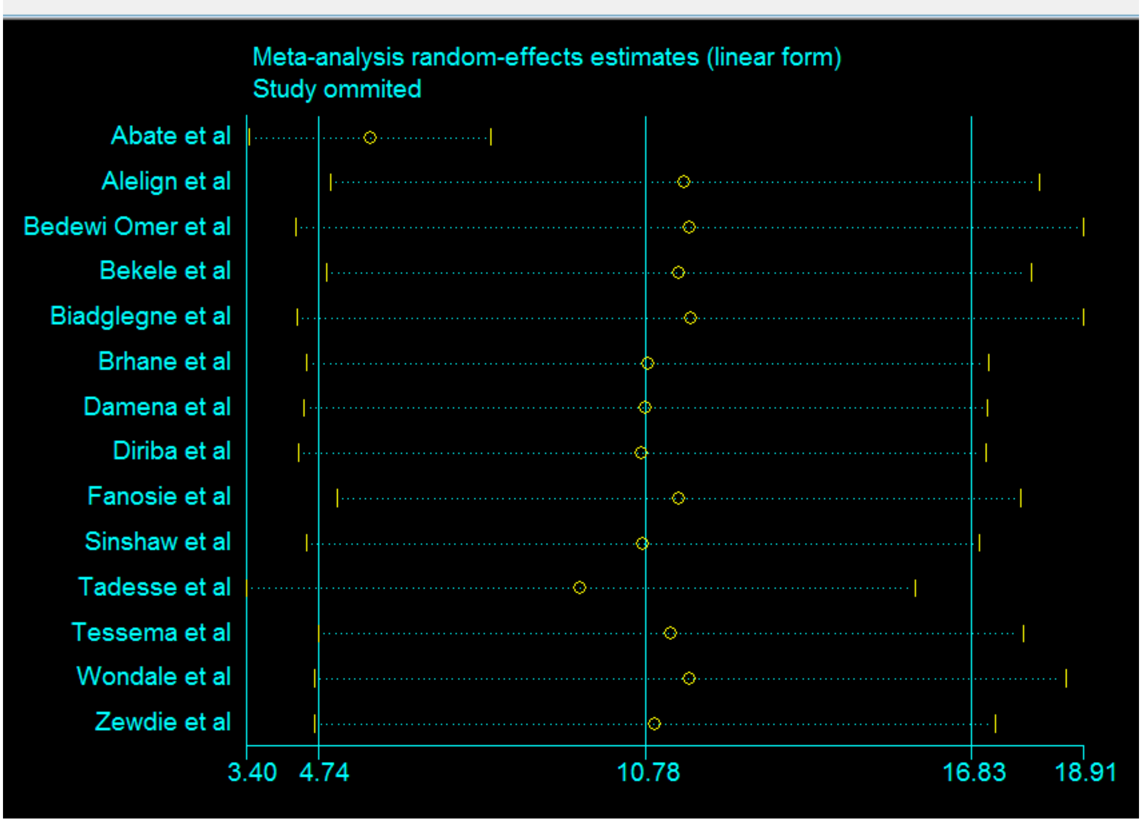

Figure S8D. Results of a sensitivity analysis assessing the between-study heterogeneity in estimating the weighted pooled prevalence of MDR-TB among overall TB cases.
