# Supplementary material for: Mycobacterium tuberculosis Drug Resistance in Ethiopia: An Updated Systematic Review and Meta-Analysis
Source: Trop Med Infect Dis. 2022 Oct 14;7(10):300. doi: 10.3390/tropicalmed7100300 (PMC9611116; doi:10.3390/tropicalmed7100300)
Supplement: Supplementary file 1 [file tropicalmed-07-00300-s001.zip › tropicalmed-1879113-Table S2.pdf]

Table S2. The search strategy used for one database (Medline/PubMed) (01 Jan 2011 to 30 Nov 2020).

MEDLINE/PUBMED			
**Group**	**Search Terms/Phrases**		
	**MeSH (Sub-terms in MeSH)**	***Non-MeSH***	**Citations**
#1	*Mycobacterium tuberculosis*<br>Tuberculosis		
#2	Drug resistance<br>Rifampicin<br>Isoniazid<br>Ethambutol<br>Pyrazinamide<br>Antitubercular agents<br>Tuberculosis, Multidrug-resistant<br>Extensively Drug-Resistant Tuberculosis	Drug susceptibility<br>Anti-tuberculosis resistance<br>Rifampicin-resistant tuberculosis<br>Isoniazid-resistant tuberculosis<br>Ethambutol-resistant tuberculosis<br>Pyrazinamide-resistant tuberculosis<br>Drug-resistant tuberculosis<br>Drug resistance pattern<br>Mono-resistance<br>Hetero resistance<br>First-line antitubercular drugs<br>Second-line antitubercular drugs	
#3		Molecular drug resistance diagnostics<br>Molecular drug susceptibility testing<br>Molecular detection of drug resistance<br>Molecular characterization of *Mycobacterium tuberculosis*<br>Genotypic drug sensitivity<br>Line-probe assay<br>MTBDR*plus* assay<br>MTBDR*plus* VER 2.0<br>GenoType®MTBDR*plus*<br>GenoType MTBDR*plus* V.2<br>Xpert MTB/RIF assay<br>GeneXpertMTB/RIF assay<br>GenoType® MTBDR*sl* assay	973
#4		The magnitude of gene mutations<br>Gene mutation conferring-drug resistance<br>Drug resistance-conferring mutations<br>Antitubercular drug resistance determinants<br>Antitubercular drug resistance mechanisms	
#5		Ethiopia	

(*Mycobacterium tuberculosis*[MeSH Terms]) OR Tuberculosis[MeSH Terms]) AND Drug resistance[MeSH Terms]) OR Drug susceptibility) OR Rifampicin[MeSH Terms]) OR Isoniazid[MeSH Terms]) OR Ethambutol[MeSH Terms]) OR Pyrazinamide[MeSH Terms]) OR Antitubercular agents[MeSH Terms]) OR Mono-resistance) OR Hetero-resistance) OR Anti-tuberculosis resistance) OR Rifampicin-resistant tuberculosis) OR Isoniazid-resistant tuberculosis) OR Ethambutol-resistant tuberculosis) OR Pyrazinamide-resistant tuberculosis) OR Drug-resistant tuberculosis) OR Drug resistance pattern) OR Tuberculosis, Multidrug-resistant[MeSH Terms]) OR Extensively Drug-Resistant Tuberculosis[MeSH Terms]) OR First-line antitubercular drugs) OR Second-line antitubercular drugs) AND Molecular diagnosis) OR Molecular detection) OR Molecular characterization) OR Molecular drug resistance diagnostics) OR Molecular drug susceptibility testing) OR Molecular detection of drug resistance) OR Molecular characterization of *Mycobacterium tuberculosis*) OR Genotypic drug sensitivity) OR Line-probe assay) OR MTBDRplus VER 2.0) OR GenoType®MTBDRplus) OR GenoType MTBDRplus V.2) OR Xpert MTB/RIF assay) OR GeneXpertMTB/RIF assay) OR GenoType® MTBDRsl assay) AND Gene mutations) OR Resistance determinants) OR Resistance mechanisms) OR Magnitude of gene mutations) OR Gene mutation conferring-drug resistance) OR Drug resistance-conferring mutations) OR Antitubercular drug resistance determinants) OR Antitubercular drug resistance mechanisms) AND Ethiopia.
