# Supplementary material for: Mycobacterium tuberculosis Drug Resistance in Ethiopia: An Updated Systematic Review and Meta-Analysis
Source: Trop Med Infect Dis. 2022 Oct 14;7(10):300. doi: 10.3390/tropicalmed7100300 (PMC9611116; doi:10.3390/tropicalmed7100300)
Supplement: Supplementary file 1 [file tropicalmed-07-00300-s001.zip › tropicalmed-1879113-Table S3.pdf]

Table S3. Quality appraisal results of included studies; Using Joanna Briggs Institute (JBI) quality appraisal checklist/protocol for cross-sectional studies [1].

Authors' name	Publication year	Evaluation Criteria's								
		1) Were inclusion criteria clearly defined?	2) Details of study subjects and the setting/s?	3) Reliable/valid measurement for exposure	4) Standard measurement for the condition	5) Were confounding factors identified?	6) Were strategies to deal with cofounding factors stated?	7) Reliable/valid measurement for outcome	8) Appropriate Statistical analysis	Quality items met (n/8),
Abate *et al.*, [2]	2014	YES	YES	YES	YES	UN	NA	YES	YES	6/8
Amir Alelign *et al.*, [3]	2019	YES	YES	YES	YES	UN	NA	YES	YES	6/8
Bedewi Omer *et al.*, [4]	2016	YES	YES	YES	YES	UN	NA	YES	YES	6/8
Bekele *et al.*, [5]	2018	YES	YES	YES	YES	UN	NA	YES	YES	6/8
Biadglegne *et al.*, [6]	2014	YES	YES	YES	YES	UN	NA	YES	YES	6/8
Biadglegne *et al.*,[7]	2013	YES	YES	YES	YES	UN	NA	YES	YES	6/8
Brhane *et al.*, [8]	2017	YES	YES	YES	YES	UN	YES	YES	YES	7/8
Damena *et al.*, [9]	2019	YES	YES	YES	YES	UN	NA	YES	YES	6/8
Diriba *et al.*, [10]	2019	YES	YES	YES	YES	YES	NA	YES	YES	7/8
Ejeta *et al.*, [11]	2018	YES	YES	YES	YES	UN	NA	YES	YES	6/8
Fanosie *et al.*, [12)	2016	YES	YES	YES	YES	UN	YES	YES	YES	7/8
Gebrehiwet *et al.*, [13]	2019	YES	YES	YES	YES	UN	NA	YES	YES	6/8
Gizachew *et al.*, [14]	2017	YES	YES	YES	YES	UN	NO	YES	YES	6/8
Habte *et al.*, [15]	2016	YES	YES	YES	YES	UN	NA	YES	YES	6/8
Haile *et al.*,[16]	2020	YES	YES	YES	YES	UN	NA	YES	YES	6/8
Jaleta *et al.*, [17]	2017	YES	YES	YES	YES	UN	NA	YES	YES	6/8
Mulu *et al.*, [18]	2017	YES	YES	YES	YES	UN	NO	YES	YES	6/8
Sinshaw *et al.*, [19]	2019	YES	YES	YES	YES	UN	YES	YES	YES	7/8
Tadesse *et al.*, [20]	2017	YES	YES	YES	YES	UN	NA	YES	YES	6/8
Tadesse *et al.*, [21]	2016	YES	YES	YES	YES	UN	NA	YES	YES	6/8
Tessema *et al.*, [22]	2012	YES	YES	YES	YES	UN	NA	YES	YES	6/8
Wondale *et al.*, [23]	2018	YES	YES	YES	YES	UN	NA	YES	YES	6/8
Workalemahu *et al.*, [24]	2013	YES	YES	YES	YES	UN	NA	YES	NO	5/8
Zewdie *et al.*, [25]	2018	YES	YES	YES	YES	UN	NA	YES	YES	6/8

NA: Not applicable, UN: Unclear.

References

1. Joanna Briggs Institute. The Joanna Briggs Institute Critical Appraisal tools for use in JBI Systematic Reviews: Checklist for Prevalence Studies. *Retre. Nov.* **2017**, *15*, 2018.

2. Abate, D., Tedla, Y., Meressa, D., Ameni, G. Isoniazid and rifampicin resistance mutations and their effect on second-line anti-tuberculosis treatment. *Int. J. Tuberc. Lung Dis*. **2014**, *18*, 946–951. http://dx.doi.org/10.5588/ijtld.13.0926.
3. Alelign, A., Zewude, A., Temesgen M., Samuel T., Gobena A., Beyene P. Molecular detection of Mycobacterium tuberculosis sensitivity to rifampicin and isoniazid in South Gondar Zone, northwest Ethiopia. *BMC Infect. Dis.* **2019**, *19*, 343.
4. Bedewi O. Z., Mekonnen Y., Worku A., Zewde A., Medhin G., Mohammed, T. Evaluation of the GenoType MTBDRplus assay for detection of rifampicin- and isoniazid-resistant Mycobacterium tuberculosis isolates in central Ethiopia. *Int. J. Mycobacteriol.* **2016**, *5*, 475–481. http://dx.doi.org/10.1016/j.ijmyco.2016.06.005.
5. Bekele S., Derese Y., Hailu E., Mihret A., Dagne K., Yamuah L. Line-probe assay and molecular typing reveal a potential drug resistant clone of Mycobacterium tuberculosis in Ethiopia. *Tropical Diseases, Travel Med. Vaccines.* **2018**, *4*, 15. https://doi.org/0.1186/s40794-018-0075-3.
6. Biadglegne F., Mulu A., Rodloff A.C., Sack, U. Diagnostic performance of the Xpert MTB/RIF assay for tuberculous lymphadenitis on fine needle aspirates from Ethiopia. *Tuberculosis (Edinb).* **2014**, *94*, 502–505. http://dx.doi.org/10.1016/j.tube.2014.05.002.
7. Biadglegne F., Tessema B., Rodloff A.C., Sack, U. Magnitude of gene mutations conferring drug resistance in mycobacterium tuberculosis isolates from lymph node aspirates in ethiopia. *Int. J. Med. Sci*. **2013**, *10*, 1589–1594. doi: 10.7150/ijms.6806.
8. Brhane, M., Kebede, A., Petros, Y. Molecular detection of multidrug-resistant tuberculosis among smear-positive pulmonary tuberculosis patients in Jigjiga town, Ethiopia. *Infect. Drug. Resist*. **2017**, *10*, 75. http://dx.doi.org/10.2147/IDR.S127903.
9. Damena D., Tolosa S., Hailemariam M., Zewude A., Worku A., Mekonnen, B. Genetic diversity and drug susceptibility profiles of Mycobacterium tuberculosis obtained from Saint Peter's TB specialized Hospital, Ethiopia. *PLoS ONE.* **2019**, *14*, e0218545. https://doi.org/10.1371/journal.pone.
10. Diriba G., Kebede A., Tola H.H., Alemu A., Tadesse M., Tesfaye, E. Surveillance of drug resistance tuberculosis based on reference laboratory data in Ethiopia. *Infect. Dis. Poverty*. **2019**, *8*, 54.
11. Ejeta E., Beyene G., Bonsa Z., Abebe, G. Xpert MTB/RIF assay for the diagnosis of Mycobacterium tuberculosis and Rifampicin resistance in high Human Immunodeficiency Virus setting in Gambella regional state, southwest Ethiopia. *J. Clin. Tuberculosis Other Mycobacterial Dis.* **2018**, *12*, 14–20.
12. Fanosie A., Gelaw B., Tessema B., Tesfay W., Admasu A., Yitayew, G. Mycobacterium tuberculosis Complex and HIV Co-Infection among Extrapulmonary Tuberculosis Suspected Cases at the University of Gondar Hospital, Northwestern Ethiopia. *PLoS ONE*. **2016**, *11*, e0150646.
13. Gebrehiwet G.B., Kahsay A.G., Welekidan L.N., Hagos A.K., Abay G.K., Hagos D.G. Rifampicin resistant tuberculosis in presumptive pulmonary tuberculosis cases in Dubti Hospital, Afar, Ethiopia. *J. Infect. Dev. Ctries*. **2019**, *13*, 21–27.
14. Gizachew Beza M., Hunegnaw E., Tiruneh, M. Prevalence and Associated Factors of Tuberculosis in Prisons Settings of East Gojjam Zone, Northwest Ethiopia. *Int. J. Bacter***.** **2017**, 3826980.
15. Habte D., Melese M., Hiruy N., Gashu Z., Jerene D., Moges F. The additional yield of GeneXpert MTB/RIF test in the diagnosis of pulmonary tuberculosis among household contacts of smear positive TB cases. *Int. J. Infect. Dis.* **2016**, *49*, 179–84.
16. Haile B., Tafess K., Zewude A., Yenew, B., Siu, G., Ameni, G. Spoligotyping and drug sensitivity of Mycobacterium tuberculosis isolated from pulmonary tuberculosis patients in the Arsi Zone of southeastern Ethiopia. *New Microbes. New Infect.* **2020**, *33*, 100620. https://doi.org/10.1016/j.nmni.2019.
17. Jaleta K.N., Gizachew M., Gelaw B., Tesfa H., Getaneh, A., Biadgo, B. Rifampicin-resistant Mycobacterium tuberculosis among tuberculosis-presumptive cases at University of Gondar Hospital, northwest Ethiopia. *Infect. Drug Resist*. **2017**, *10*, 185.
18. Mulu W., Abera B., Yimer M., Hailu, T., Ayele, H., Abate, D. Rifampicin-resistance pattern of Mycobacterium tuberculosis and associated factors among presumptive tuberculosis patients referred to Debre Markos Referral Hospital, Ethiopia: a cross-sectional study. *BMC Research Notes*. **2017**, *10,* 8.
19. Sinshaw W., Kebede A., Bitew A., Tesfaye E., Tadesse M., Mehamed, Z. Prevalence of tuberculosis, multidrug resistant tuberculosis and associated risk factors among smear negative presumptive pulmonary tuberculosis patients in Addis Ababa, Ethiopia. *BMC Infect. Dis.* **2019**, *19*, 641.
20. Tadesse M., Abebe G., Bekele A., Bezabih M., de Rijk P., Meehan C.J. The predominance of Ethiopian specific Mycobacterium tuberculosis families and minimal contribution of Mycobacterium bovis in tuberculous lymphadenitis patients in Southwest Ethiopia. *Infect. Gene. Evolut*. **2017**, *55*, 251–259. doi: 10.1016/j.meegid.2017.09.016.
21. Tadesse M., Aragaw D., Dimah B., Efa F., Abdella K., Kebede W. Drug resistance-conferring mutations in Mycobacterium tuberculosis from pulmonary tuberculosis patients in Southwest Ethiopia. *Int. J. Mycobacteriol.* **2016**, *5*, 185–191. http://dx.doi.org/10.1016/j.ijmyco.2016.02.009.
22. Tessema B., Beer J., Emmrich F., Sack, U., Rodloff A.C. Analysis of gene mutations associated with isoniazid, rifampicin and ethambutol resistance among Mycobacterium tuberculosis isolates from Ethiopia. *BMC Infect. Dis.* **2012**, *12*, 37. doi:10.1186/471-2334-12-37.

23. Wondale B., Medhin G., Abebe G., Tolosa S., Mohammed T., Teklu T. Phenotypic and genotypic drug sensitivity of Mycobacterium tuberculosis complex isolated from South Omo Zone, Southern Ethiopia. *Infect. Drug. Resist.* **2018**, *11*, 1581–1589. http://dx.doi.org/10.2147/IDR.S165088.
24. Workalemahu B., Berg S., Tsegaye W., Abdissa A., Girma T., Abebe M. Genotype diversity of Mycobacterium isolates from children in Jimma, Ethiopia. *BMC Res. Notes.* **2013**, *6*, 352. doi:10.1186/756-0500-6-352.
25. Zewdie O., Mihret A., Abebe T., Kebede A., Desta K., Worku A. Genotyping and molecular detection of multidrug-resistant Mycobacterium tuberculosis among tuberculosis lymphadenitis cases in Addis Ababa, Ethiopia. *New Microbes. New Infect.* **2018**, *21*, 36–41. https://doi.org/10.1016/j.nmni.2017.10.009.
